# Loneliness and cognition in older adults: A meta-analysis of harmonized studies from the United States, England, India, China, South Africa, Mexico, and Chile

**DOI:** 10.1017/S003329172500011X

**Published:** 2025-02-20

**Authors:** Ji Hyun Lee, Angelina R. Sutin, André Hajek, Selin Karakose, Damaris Aschwanden, Páraic S. O’Súilleabháin, Yannick Stephan, Antonio Terracciano, Martina Luchetti

**Affiliations:** 1 Department of Human Development and Community Health, Montana State University, Bozeman, MT, USA; 2 Department of Behavioral Sciences and Social Medicine, College of Medicine, Florida State University, Tallahassee, FL, USA; 3 Department of Geriatrics, College of Medicine, Florida State University, Tallahassee, FL, USA; 4 Department of Health Economics and Health Services Research, University Medical Center Hamburg-Eppendorf, Hamburg, Germany; 5 Center for the Interdisciplinary Study of Gerontology and Vulnerability, University of Geneva, Geneva, Switzerland; 6 Department of Psychology, University of Limerick, Limerick, Ireland; 7 EuroMov, University of Montpellier, Montpellier, France

**Keywords:** cognition, loneliness, meta-analysis, older adults

## Abstract

**Background:**

Loneliness is a risk factor for late-life dementia. There is less consistent evidence of its association with cognitive performance. This study examined the replicability of the association between loneliness and overall and domain-specific cognitive function and informant-rated cognitive decline in cohorts from seven countries: the United States, England, India, China, South Africa, Mexico, and Chile.

**Methods:**

Data were from the Harmonized Cognitive Assessment Protocol administered in seven population-based studies (total N > 20,000). Participants reported their loneliness, completed a battery of cognitive tests, and nominated a knowledgeable informant to rate their cognitive decline. Random-effect meta-analyses were used to summarize the associations from each cohort.

**Results:**

Loneliness was associated with poor overall cognitive performance and informant-rated cognitive decline controlling for sociodemographic factors (meta-analytic correlation for overall cognition = −.10 [95% CI = −.13, −.06] and informant-rated decline = .16 [95% CI = .14, .17]). Despite some heterogeneity, the associations were significant across samples from Africa, Asia, Europe, North, Central, and South America. The meta-analysis also indicated an association with specific cognitive domains: episodic memory, speed-attention, visuospatial abilities, numeric reasoning, and verbal fluency. The associations were attenuated but persisted when depressive symptoms were added as a covariate. Depression, cognitive impairment, and sociodemographic factors did not consistently moderate the associations across samples.

**Conclusions:**

Loneliness is associated with poor performance across multiple domains of cognition and observer-rated cognitive decline, associations that replicated across diverse world regions and cultures.

The World Health Organization (WHO) have called attention to the public health crisis of loneliness (WHO, 2021, 2023). About three in ten older adults feel lonely worldwide (Susanty et al., 2025), with widespread, documented consequences for their health and well-being (National Academies of Sciences, Engineering, and Medicine, 2020). Feeling lonely is the subjective perception that one does not have their desired social connections, regardless of whether alone or not (Hawkley & Cacioppo, 2010). As such, it is related but distinct from being isolated or alone. Such feelings are associated with premature mortality (Wang et al., 2023) and increased risk for cardiovascular problems, immune dysfunctions, impaired sleep, and depressive symptoms (Cacioppo, Hawkley, & Thisted, 2010; Christiansen, Larsen, & Lasgaard, 2016; Steptoe, Owen, Kunz-Ebrecht, & Brydon, 2004), all factors important for cognitive health. Feeling lonely is associated with an increased risk of all-cause dementia, including Alzheimer’s disease and vascular dementia, and cognitive impairment (Luchetti et al., 2024). There is less consistent evidence for the relation between loneliness and cognitive function, which impacts everyday life, even before the onset of impairment.

In a recent meta-analysis, Harrington et al. (2023) pooled data from six independent studies (n = 10,954; age > 60 years) and identified a significant cross-sectional association between loneliness and global cognitive function (r = − .08, 95% CI = −.14, −.02). The authors noted large heterogeneity across studies (I^2^ = 84) and a lack of sufficient data to examine associations with specific cognitive domains in a meta-analysis. In studies that have examined episodic memory (e.g., word recall), the association has been inconsistent. Some researchers find a negative association, both cross-sectionally and longitudinally (Desai et al., 2023; Estrella et al., 2021; Kang et al., 2024; Souza et al., 2023; Tao et al., 2022), whereas others have not found an association between loneliness and memory (Samtani et al., 2022; Sol, Sharifian, Manly, Brickman, & Zahodne, 2021; Solé-Padullés et al., 2022), or reported a significant association only for some participants (e.g., adults over 65) but not others (Cachón-Alonso, Hakulinen, Jokela, Komulainen, & Elovainio, 2023). Results are also mixed in studies that examined executive function or performance on speed-attention tasks: Some studies reported a negative association (Desai et al., 2023; Estrella et al., 2021; Samtani et al., 2022; Tao et al., 2022), but others not (Kyröläinen & Kuperman, 2021; McVeigh et al., 2024; Windsor, Ghisletta, & Gerstorf, 2020). Similarly, mixed associations are observed with poor verbal fluency, a marker of subsequent cognitive impairment (Sutin, Stephan, & Terracciano, 2019b), with studies reporting both significant (Cachón-Alonso et al., 2023; Estrella et al., 2021; Souza et al., 2023) and non-significant associations (Kyröläinen & Kuperman, 2021; Windsor et al., 2020).

These findings suggest that loneliness may have a negative association with cognitive function across multiple domains. There are methodological differences—from the different tasks administered, to sample size, location, and composition, to selection of covariates—that limit comparability across studies. The present study expands the literature on loneliness and cognitive health in several ways. First, it uses data from the Harmonized Cognitive Assessment Protocol (HCAP) administered in the Health and Retirement Study (HRS) and six HRS-sister studies. The use of HCAP increases comparability across studies to examine the association between loneliness and cognitive function across five domains (Sutin, Stephan, Luchetti, & Terracciano, 2019a; Weir, Langa, & Ryan, 2016): episodic memory; attention and processing speed; visuospatial abilities; numeric reasoning; and verbal fluency. Second, HCAP also collects information on cognitive decline reported by a knowledgeable informant. While there is evidence of an association between loneliness and self-reported cognitive decline (Reynolds et al., 2022), to our knowledge, no study has examined whether this association extends to cognitive decline observed by knowledgeable others. This measure is important because close others may be able to detect cognitive deficits that occur in daily life that cannot be detected with standardized cognitive tests (Pérez-Blanco et al., 2022). Third, with few exceptions (Foong, Ibrahim, Abdullah, & Bagat, 2024; Kyaw & Levine, 2023; Souza et al., 2023), most published work has tested samples from North America and Europe, which limits generalizability of the associations to other countries and cultures. With the use of seven samples with harmonized measures across the world, this study addresses the lack of data from geographic areas, particularly from Africa and South America.

Based on prior research, we expect loneliness to be associated with poor overall cognitive performance and informant-rated cognitive decline. We do not make specific hypotheses for the cognitive domains because of the mixed associations reported in the literature. In addition, we examined whether the associations were moderated by cognitive status (impaired versus unimpaired) and depressive symptomatology because both factors are relevant for loneliness (Carrasco et al., 2024; Susanty et al., 2025) and impair performance on cognitive tasks (Henry, Crawford, & Phillips, 2004; Semkovska et al., 2019). We also examined whether the association varied by sociodemographic factors (age, sex, education, marital status, living arrangements, and race and/or ethnicity [where possible]) to examine generalizability of the associations across sociodemographic groups. We performed the analysis separately in each HCAP study and then combined the results in a meta-analysis.

## Methods

### Participants

Participants were from seven cohort studies that administered HCAP: the HRS, the English Longitudinal Study of Ageing (ELSA), the Diagnostic Assessment of Dementia for the Longitudinal Aging Study in India (LASI-DAD), the China Health and Retirement Longitudinal Study (CHARLS), the Dementia Study of the Health and Aging in Africa (DS-HAALSI), the Mexican Cognitive Aging Ancillary Study (Mex-Cog) of the Mexican Health and Aging Study (MHAS), and the Chile Cognitive Aging Study (Chile-Cog). Data were from the wave that the HCAP protocol was administered for the first time, except for Mex-Cog, for which we used the wave in which HCAP was administered in the same year as of the regular assessment. Detailed information about measures, administration, and scoring can be found in Weir et al. (2016), Sutin and colleagues (Sutin, Luchetti, Stephan, & Terracciano, 2024; Sutin, Stephan, Luchetti, et al., 2019) and Supplementary Material (Table S1-S3).

Of the HRS participants, those who completed the 2016 interview and were 65 and over were eligible for HCAP. Of the eligible participants, a random subset was invited to participate in HCAP (n = 5,500). Of those, 3,496 completed at least part of the protocol between June 2016 and October 2017. Participants could have the HCAP assessment administered in either English or Spanish. Loneliness was measured in the 2014 or 2016 regular assessment; the sociodemographic covariates were from the same assessment as loneliness. The analyses included 2,829 individuals (60.1% females; age at the regular assessment M = 74.46, SD = 7.39) with data on loneliness, age, sex, education, race, ethnicity, and marital status, and at least one HCAP cognitive measure; informant ratings were available for 2,587 participants.

ELSA participants were eligible for the HCAP assessment if they were 65 or older by January 2018 and completed an in-person interview in Wave 8 (2016/2017) or Wave 7 (2014/2015). Of the eligible participants (n = 5,715), only a subset was randomly invited to complete the protocol (n = 1,778). A total of 1,273 participants completed at least part of the protocol between January 2018 and April 2018. Loneliness was from the 2016/2017 regular assessment; the covariates were from the same assessment as loneliness. The analyses included 1,064 individuals (54.0% females; age at the regular assessment M = 74.50, SD = 7.03) with data on loneliness, age, sex, education, race, and marital status, and at least one HCAP cognitive measure; informant ratings were available for 901 participants.

LASI-DAD assessed a sub-sample of 4,096 respondents from the baseline LASI sample at age 60 or older. HCAP was administered between October 2017 and March 2020 after the regular LASI interview and included a question on loneliness. The analyses included 4,021 individuals (53.9% females; age M = 69.63, SD = 7.47) with data on loneliness, age, sex, education, and marital status, and at least one HCAP cognitive measure; informant ratings were available for 3,963 participants.

CHARLS used HCAP to pilot cognitive tasks to include in their regular interview. Participants aged 60 and older (n = 11,021) and their informants were selected to complete part of the HCAP in Wave 4 (2018). The analyses included 9,588 individuals (50.3% females; age M = 68.07, SD = 6.48) with data on loneliness, age, sex, education, and marital status, and at least one HCAP cognitive measure; informant ratings were available for 8,837 participants.

DS assessed a subsample of HAALSI participants aged 50 and older. HCAP was administered to 635 respondents between September 2019 and January 2020 and included a question on loneliness; covariates were from the main HAALSI survey administered in 2018–19. The analyses included 630 individuals (61.7% females; age at the main survey, M = 69.17, SD = 11.56) with data on loneliness, age, sex, education, and marital status, and at least one HCAP cognitive measure; informant ratings were available for 625 participants.

Mex-Cog assessed cognitive function in a subsample of MHAS participants aged 58 and older in 2021. For the current analysis, information on loneliness was from the regular interview conducted in the same year as the HCAP assessment. Of the 4,066 participants selected, 3,575 completed at least part of the assessment. The analyses included 3,198 individuals (57.2% females; age M = 72.19, SD = 7.49) with data on loneliness, age, sex, education, and marital status, and at least one HCAP measure; informant ratings were available for 3,172 participants.

Chile-Cog assessed cognitive function among Chilean adults aged 60 and older as part of the Chilean Social Protection Survey. A total of 2,033 participants were tested in 2019. The analyses were based on 1,967 individuals (57.1% females; age M = 70.89, SD = 8.11) with data on loneliness, age, sex, and at least one HCAP cognitive measure (we were unable to retrieve information on other sociodemographic factors at the time of analysis); informant ratings were available for 1,712 participants.

### Measures

**Loneliness.** HRS, ELSA, Mex-Cog, and Chile-Cog included three items from the University of California, Los Angeles (UCLA) Loneliness Scale (Hughes, Waite, Hawkley, & Cacioppo, 2004). Respondents were asked how often they “lack companionship,” “felt left out/ignored,” and “isolated from others.” Responses were on a 3-point scale, from *often* to *hardly ever or never.* Chile-Cog used a 4-point scale (*never*, *rarely*, *sometimes*, and *always*). Items were reverse scored in the direction of loneliness when appropriate and the average taken across items (alphas ranged from .73 in Chile-Cog to .84 in ELSA). Except for Chile-cog, the scale was administered in the main interview; the pattern of results was the same controlling for time between the loneliness assessment and HCAP administration. For CHARLS, LASI-DAD, and HAALSI, loneliness was assessed with a single item, “In the past week… I felt lonely,” from the Center for Epidemiologic Studies Depression (CES-D) scale (e.g., Karim, Weisz, Bibi, & ur Rehman, 2015), administered with the HCAP measures; responses were on a 4-point scale, from *rarely or none (<1 day)* to *most of the time (5–7 days).*

**Cognitive performance.** The cognitive tasks in HCAP were grouped into five domains (Sutin, Stephan, Luchetti, et al., 2019; Weir et al., 2016): (1) *Episodic memory* was measured in most samples with three tasks: the CERAD Word List Learning and Recall Task (immediate, delayed, recognition) and a version of the Wechsler Memory Scale – Logical Memory I, Long Story (immediate, delayed, recognition), and/or the Brave Man, Short Story (immediate, delayed). (2) *Speed-attention* was measured with the following tests: Letter or Symbol Cancellation, the Symbol-Digit Modalities Test, Backward Naming or Counting, and/or Trail Making A and B. (3) *Visuospatial abilities* were measured with Constructional Praxis (immediate and recall) and/or Raven’s matrices. (4) *Numeric reasoning* was measured with the HRS Number Series. (5) A *verbal fluency* task (animal category). In addition, global cognitive function was measured with the Mini-Mental State Examination (MMSE; Folstein, Folstein, & Fanjiang, 2001). Each task was scored and standardized. For domains with multiple tasks, the mean was taken across standardized scores. Trails A and B were reversed (multiplied by −1) to match the scoring direction of other speed-attention measures. Supplementary Table S1 describes task variations across studies.

**Informant-rated cognitive decline.** For each participant, one person who knew them well completed informant-rated measures of cognitive decline. Studies included at least one of the following measures: the Informant Questionnaire on Cognitive Decline in the Elderly (IQ-CODE; Jorm, 1994), the Community Screening Instrument for Dementia (CSI-D; Hall et al., 2000), the Blessed Dementia Rating Scale Part I (Morris et al., 1989), and the 10/66 (Prince et al., 2011). These scales were scored and standardized, and the mean taken in the direction of greater informant-perceived decline. Each scale and variation across studies are described in Supplementary Table S2.

**Covariates and moderators.** Covariates were age (years), sex (1 = female, 0 = male), education (reported in years or as the highest level of education), marital status (1 = married or cohabitating with a partner, 0 = single, separated, divorced or widowed), race (1 = Black/other or non-White, 0 = White for HRS and ELSA) and ethnicity (1 = Hispanic/Latinx, 0 = non-Hispanic/Latinx for HRS). Administration could be in more than one language within a country (e.g., HRS) or with the help of a translator in others (e.g., CHARLS). For informant-rated cognitive decline, we controlled for characteristics of the informant and their relationship with the respondent: informant age, sex (1 = female, 0 = male), education, whether the informant was the spouse (1 = yes, 0 = no), and whether they lived with the respondent (1 = yes, 0 = no). Additional analysis further controlled for depressive symptoms, measured with the CES-D (excluding the loneliness item), and living alone, as a proxy for isolation (1 = living alone, 0 = with others). Depressive symptoms and severe cognitive impairment, defined as 1.5 SD below the sample mean on the MMSE, were tested as moderators, as both are associated with loneliness (Susanty et al., 2025) and cognition (Henry et al., 2004; Semkovska et al., 2019). Covariates and moderators are described in Supplementary Table S3.

### Statistical analysis

Linear regressions were used to examine the association between loneliness and the cognitive measures in each sample using SPSS (Version 29). The score of each cognitive domain was predicted by loneliness, controlling for age, sex, education, marital status, race and ethnicity (where available). All continuous predictors were standardized, and dichotomous predictors were coded 0/1 to facilitate comparison across samples. Language of administration and use of a translator were also assessed as covariates in HRS, LASI-DAD, and CHARLS, but were not included in the final models because they did not impact the results. For scores based on performance on multiple cognitive tasks, supplemental analyses examined the association between loneliness and each task to determine whether a specific task was driving domain-level associations. Follow-up analyses tested whether associations held controlling for depressive symptoms and living alone, respectively. We repeated the same strategy for the informant ratings of cognitive decline, controlling for both participant characteristics and informant and relationship characteristics.

**Moderation.** Additional analyses tested if associations varied by depressive symptoms or cognitive impairment, examining interactions between loneliness and these factors. Depressive symptomatology was tested as a continuous moderator, and cognitive impairment was tested as a dichotomous moderator. We also tested whether age, sex, education, marital status, living alone, and race/ethnicity (where possible) moderated the associations in each sample.

**Meta-analysis.** Results from the individual samples were summarized with random-effect meta-analyses using STATA (StataNow 18.5 SE). Sub-group analyses were used to test whether the association between loneliness and each cognitive domain and informant-rated decline varied by type of loneliness measure (1-item versus 3-item scale) and country (United States/England versus others). Interaction terms for the moderation analyses were also meta-analyzed to summarize their effect across samples.

All associations were reported with standardized beta coefficients, which can be interpreted as an effect size. For interpretation, we focused on p-values <.01 due to the large number of analyses.

## Results

**Figure 1.**
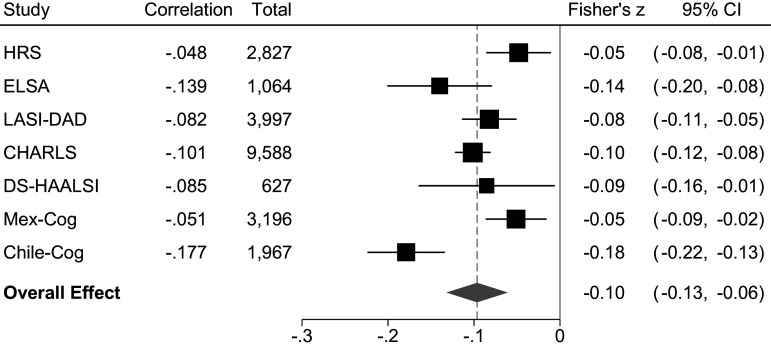
Loneliness association with overall cognitive function. Studies are the Health and Retirement Study (HRS), the English Longitudinal Study of Ageing (ELSA), the Diagnostic Assessment of Dementia for the Longitudinal Aging Study in India (LASI-DAD), China Health and Retirement Longitudinal Study (CHARLS), Dementia Study of the Health and Aging in Africa (DS-HAALSI), the Mexican Cognitive Aging Ancillary Study (Mex-Cog) of the Mexican Health and Aging Study, and the Chile Cognitive Aging Study (Chile-Cog). Black boxes represent the correlation for each study; the size of each box indicates the influence of the correlation on the model. The solid gray line indicates a correlation of zero. The dotted line and the diamond indicate the meta-analytic association. The analysis accounted for age, sex, education, marital status, and race and ethnicity (where possible). Supplementary Table S5 and S6 report results when further accounting for depressive symptoms and living arrangements.

**Figure 2.**
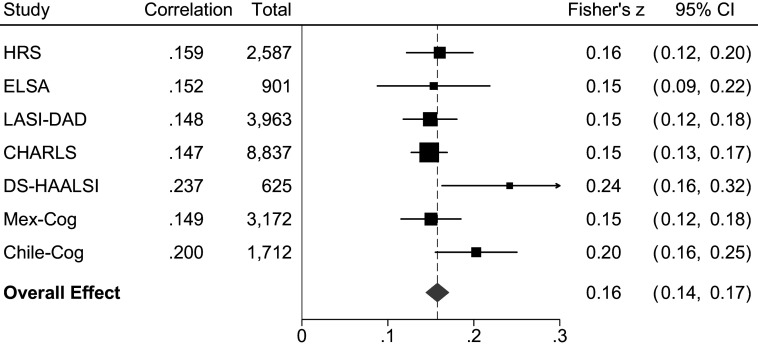
Loneliness association with informant ratings of cognitive decline. Studies are the Health and Retirement Study (HRS), the English Longitudinal Study of Ageing (ELSA), the Diagnostic Assessment of Dementia for the Longitudinal Aging Study in India (LASI-DAD), China Health and Retirement Longitudinal Study (CHARLS), Dementia Study of the Health and Aging in Africa (DS-HAALSI), the Mexican Cognitive Aging Ancillary Study (Mex-Cog) of the Mexican Health and Aging Study, and the Chile Cognitive Aging Study (Chile-Cog). Black boxes represent the correlation for each study; the size of each box indicates the influence of the correlation on the model. The solid gray line indicates a correlation of zero. The dotted line and the diamond indicate the meta-analytic association. The analysis accounted for age, sex, education, marital status, and race and ethnicity (where possible). Supplementary Table S5 and S6 report results when further accounting for depressive symptoms and living arrangements.

Descriptive statistics for each sample are in Table 1 and Supplementary Table S4. As shown in Figures 1 and 2, loneliness was consistently associated with poor overall performance and with informant ratings of cognitive decline in each of the seven countries. The meta-analytic effects were − .10 (95%CI = −.13, −.06; N = 23,266) for overall cognition and .16 (95% CI = .14, .17; N = 21,797) for informant-rated decline. The associations were similar in cohorts where loneliness was measured with 1-item (n = 3) versus 3-item (n = 4); there were no statistically significant differences between the United States/England versus other countries (see Supplementary Figures S1 and S2). There was significant heterogeneity in the association between loneliness and overall cognition (Q = 29.32, p < .01; I^2^ = 83.83), which suggested differences in the magnitude of the association across studies, which ranged from −.05 in the United States and Mexico to −.18 in Chile. No significant heterogeneity was observed for the association with informant-rated decline (Q = 9.17, p > .05; I^2^ = 3.57). When considering results from models that accounted for depressive symptoms, the meta-analytic effect of loneliness remained significant, but it was reduced by about 60% (Supplementary Table S5)—i.e., −.04 (95% CI = −.05, −.03) for overall cognitive performance (3 out of 7 studies with an association at p < .01) and .07 (95% CI = .03, .10) for informant ratings of decline (4 out of 7 studies with an association at p < .01). The associations held across samples when additionally controlling for living arrangements (Table S6).

**Table 1.** Descriptive statistics for each sample

	HRS	ELSA	LASI-DAD	CHARLS	DS-HAALSI	Mex-Cog	Chile-Cog
N	2,829	1,064	4,021	9,588	630	3,198	1,967
Country	United States	England	India	China	South Africa	Mexico	Chile
Loneliness	1.45(0.52);1–3	1.39(0.51);1–3	1.66(0.95); 1–4	1.66(1.07);1–4	0.63 (0.81);0–3	1.35(0.52);1–3	1.71(0.71);1–4
Age (years)	74.46(7.39);	74.50(7.03);	69.63(7.47);	68.07(6.48);	69.17(11.56);	72.19(7.49);	70.89(8.11);
	62–98	63–90+	60–105	56–108	48–99	58–101	60–98
Sex (females)	60.1% (1,699)	54.0% (575)	53.9% (2,167)	50.3% (4,820)	61.7% (389)	57.2% (1,830)	57.1% (1,123)
Education (years or level) ^a^	12.91(3.00)	30.4% (323)	48.7% (1,958)	52.0% (4,989)	55.1% (347)	5.34 (4.45)	--
Married/with partner	59.1% (1,671)	65.8% (700)	64.7% (2,602)	76.8% (7,364)	39.7% (250)	52.8% (1,687)	--
Living alone	26.4% (746)	30.2% (321)	4.5% (181)	12.0% (1150)	10.2% (64)	12.9% (412)	--
Black/non-white	18.8% (533)	2.4% (26)	--	--	--	--	--
Depressive symptoms	1.09(1.60);0–7	1.26(1.62);0–7	2.05(0.55);1–4	1.92(0.69);1–4	0.68(0.47);0–2.5	2.98(2.29);0–8	3.56(3.23);0–13
Cognitive impairment ^b^	8.0% (226)	7.0% (75)	7.9% (316)	9.1% (871)	7.1% (45)	8.6% (275)	11.1% (218)
**Cognitive measures**							
Overall Cognition	✓	✓	✓	✓	✓	✓	✓
Episodic Memory	✓	✓	✓	✓	✓	✓	✓
Speed-Attention	✓	✓	✓		✓	✓	✓
Visuospatial Abilities	✓	✓	✓		✓	✓	✓
Numeric Reasoning	✓	✓					
Verbal Fluency	✓	✓	✓	✓	✓	✓	✓
**Informant ratings**							
N	2,587	901	3,963	8,837	625	3,172	1,712
Informant age (years)	64.73 (14.20);	66.55 (12.80);	44.32 (16.80);	58.48 (14.83);	43.93 (18.08);	50.18 (15.42);	53.00 (16.39);
	18–96	20–90+	18–92	18–96	18–92	18–97	15–92
Informant sex (females)	67.0% (1,734)	65.0% (586)	63.9% (2,533)	53.0% (4,684)	68.2% (426)	69.7% (2,229)	71.1% (1,218)
Informant education (years or level) ^a^	13.51 (2.62)	21.8% (196)	22.6% (895)	33.7% (2,975)	11.7% (74)	4.8% (155)	24.4% (417)
Relationship (spouse)	46.7% (1,209)	58.3% (525)	30.2% (1,195)	56.1% (4,960)	27.5% (173)	28.5% (913)	34.2% (585)
Living Together	26.2% (677)	55.0% (496)	75.9% (2.999)	64.1% (5,666)	63.5% (397)	70.4% (2,251)	74.6% (1,277)

*Note:* Studies are the Health and Retirement Study (HRS), the English Longitudinal Study of Ageing (ELSA), the Diagnostic Assessment of Dementia for the Longitudinal Aging Study in India (LASI-DAD), China Health and Retirement Longitudinal Study (CHARLS), Dementia Study of the Health and Aging in Africa (DS-HAALSI), the Mexican Cognitive Aging Ancillary Study (Mex-Cog) of the Mexican Health and Aging Study, and the Chile Cognitive Aging Study (Chile-Cog). Values represent means (standard deviations) or percent (number); range. ^a^ Level corresponds to the percentage of participants with no formal/low education. ^b^ Cognitive impairment was defined as a score 1.5 SD below the sample mean on the MMSE. Cognitive scores and zero-order correlations for each measure and cognitive domain within each sample are reported in Supplementary Table S4.

The meta-analysis also indicated an association between loneliness and poor performance in specific cognitive domains (Table 2): episodic memory (3/7 studies with a negative association at p < .01; meta-analytic estimate = −.07 [95% CI = −.11, −.04]), speed-attention (6/6 studies with a negative association at p < .01; meta-analytic estimate = −.11 [95% CI = −.15, −.06]), visuospatial abilities (4/6 studies with a negative association at p < .01; meta-analytic estimate = −.08 [95% CI = −.12, −.05]), numeric reasoning (2/2 studies with a negative association at p < .01; meta-analytic estimate = −.10 [95% CI = −.13, −.06]) and verbal fluency (5/7 studies with a negative association at p < .01; meta-analytic estimate = −.06 [95% CI = −.09, −.04]). Subgroup analyses indicated no statistically significant differences related to measures of loneliness (1-item versus 3-item) or between the United States/England versus other countries in the association of loneliness with the different domains (Supplementary Material, Figures S3-S6). Except for numeric reasoning (assessed only in the United States/England), there was significant heterogeneity across studies, with I^2^ values >50, which indicated differences in the magnitude of the association across samples for most domains. The domain-level associations were generally not driven by the specific tasks used to compute the composite scores. For episodic memory, however, loneliness was more consistently related to the CERAD Word List Learning and Recall Task than other tasks (see Supplementary Table S7). The meta-analytic effect of loneliness was reduced in size but remained significant (ps < .01) when accounting for depressive symptoms (Supplementary Table S8), except for the association with verbal fluency (p = .099). The associations with each domain were similar when further controlling for living alone (Table S9).

**Table 2.** Loneliness associations with five cognitive domains

Episodic memory		Regressions		Meta-analysis	
Studies (7)	N	β	p	Fisher’s z (95% CI)	% weight
HRS	2,828	−.042	.011	−.042 (−.079, −.005)	15.30
ELSA	1,062	−.053	.060	−.053 (−.113, .007)	11.93
LASI-DAD	3,987	−.066	<.001	−.066 (−.097, −.035)	16.09
CHARLS	8,442	−.096	<.001	−.096 (−.118, −.075)	17.24
DS-HAALSI	630	−.045	.216	−.045 (−.123, .033)	9.60
Mex-Cog	3,189	−.031	.043	−.031 (−.066, .004)	15.60
Chile-Cog	1,967	−.165	<.001	−.167 (−.211, −.122)	14.24
Overall Effect		Estimate = −.073 (−.108, −.038), p < .001			
Heterogeneity		Q = 30.80, I^2^(%) = 83.19, tau^2^ = .002			

Speed-attention		Regressions		Meta-analysis	
Studies (6)	N	β	p	Fisher’s z (95% CI)	% weight
HRS	2,822	−.080	<.001	−.080 (−.117, −.043)	18.18
ELSA	1,061	−.146	<.001	−.147 (−.207, −.087)	14.90
LASI-DAD	3,905	−.065	<.001	−.065 (−.096, −.034)	18.87
DS-HAALSI	630	−.110	.001	−.110 (−.189, −.032)	12.43
Mex-Cog	3,174	−.064	<.001	−.064 (−.099, −.029)	18.45
Chile-Cog	1,957	−.191	<.001	−.193 (−.238, −.149)	17.17
Overall effect		Estimate = −.108 (−.151, −.064), p < .001			
Heterogeneity		Q = 29.07, I^2^(%) = 83.27, tau^2^ = .002			

Visuospatial abilities		Regressions		Meta-analysis	
Studies (6)	N	β	p	Fisher’s z (95% CI)	% weight
HRS	2,816	−.063	<.001	−.063 (−.100, −.026)	19.22
ELSA	1,058	−.116	<.001	−.117 (−.177, −.056)	13.50
LASI-DAD	3,935	−.089	<.001	−.089 (−.120, −.058)	20.71
DS-HAALSI	603	−.080	.024	−.080 (−.160, −.000)	9.92
Mex-Cog	3,033	−.032	.038	−.032 (−.068, .004)	19.57
Chile-Cog	1,889	−.136	<.001	−.137 (−.182, −.092)	17.08
Overall effect		Estimate = −.084 (−.116, −.052), p < .001			
Heterogeneity		Q = 15.16, I^2^(%) = 68.66, tau^2^ = .001			

Numeric reasoning		Regressions		Meta-analysis	
Studies (2)	N	β	p	Fisher’s z (95% CI)	% weight
HRS	2,456	−.087	<.001	−.087 (−.127, −.048)	71.37
ELSA	987	−.118	<.001	−.118 (−.181, −.056)	28.63
Overall effect		Estimate = −.096 (−.130, −.063), p < .001			
Heterogeneity		Q = 0.69, I^2^(%) = 0.01, tau^2^ = .000			

Verbal fluency		Regressions		Meta-analysis	
Studies (7)	N	β	p	Fisher’s z (95% CI)	% weight
HRS	2,828	−.040	.022	−.040 (−.077, −.003)	15.63
ELSA	1,062	−.078	.008	−.078 (−.138, −.018)	9.91
LASI-DAD	3,945	−.042	.006	−.042 (−.073, −.011)	17.34
CHARLS	8,181	−.064	<.001	−.064 (−.086, −.042)	20.22
DS-HAALSI	629	−.036	.369	−.036 (−.114, .042)	7.05
Mex-Cog	3,198	−.049	.003	−.049 (−.084, −.014)	16.29
Chile-Cog	1,964	−.135	<.001	−.136 (−.180, −.092)	13.56
Overall effect		Estimate = −.063 (−.088, −.038), p < .001			
Heterogeneity		Q = 14.88, I^2^(%) = 65.29, tau^2^ = .001			

*Note:* Studies are the Health and Retirement Study (HRS), the English Longitudinal Study of Ageing (ELSA), the Diagnostic Assessment of Dementia for the Longitudinal Aging Study in India (LASI-DAD), China Health and Retirement Longitudinal Study (CHARLS), Dementia Study of the Health and Aging in Africa (DS-HAALSI), the Mexican Cognitive Aging Ancillary Study (Mex-Cog) of the Mexican Health and Aging Study, and the Chile Cognitive Aging Study (Chile-Cog). N is the number of participants with data for each cognitive domain. βs are standardized coefficients from regressions within each sample. We further report Fisher’s z (95% Confidence Intervals) and weights for each study included in the meta-analysis. The analysis accounted for age, sex, education, marital status, and race and ethnicity (where possible). Supplementary Table S8 and S9 reports results when further accounting for depressive symptoms and living arrangements.

**Moderation.** The meta-analysis of the interactions indicated that only a few associations between loneliness and HCAP measures were moderated by depressive symptoms and cognitive impairment (Supplementary Table S10). The loneliness × depression interaction was significant for speed-attention (meta-analytic estimate = .04 [95% CI = .02, .06]) and verbal fluency (meta-analytic estimate = .03 [95% CI = .02, .05]), with an association observed for participants with lower depressive symptoms in India, China, and Chile (Figure S7). For episodic memory, but not other domains, the meta-analysis indicated a significant loneliness × cognitive impairment interaction (meta-analytic estimate = .02 [95% CI = .01, .03]); the association was significant among individuals without cognitive impairment in the Chinese sample (Figure S7), but the interaction did not replicate in other samples. The meta-analysis also indicated that for most measures the observed associations did not vary by sociodemographic characteristics (Table S10 and Figure S7). The exception was a significant loneliness × education interaction predicting overall cognition (meta-analytic estimate = .04 [95% CI = .02, .05]), with a slightly stronger association for individuals with lower education in samples from India, China and Mexico. There was also a significant loneliness × sex interaction predicting informant ratings (meta-analytic estimate = .05 [95% CI = .01, .08]); the association with informant-rated decline was stronger for females in the Chilean sample, but the interaction term was non-significant in the other samples.

## Discussion

This study examined the association between loneliness and multiple measures of cognitive function in seven HCAP studies and combined the results in a meta-analysis (total N > 20,000). Consistent with the literature on loneliness and risk for dementia (Luchetti et al., 2024), the meta-analysis found an association between loneliness and poor performance across cognitive tasks measuring overall cognitive function (MMSE), and specific cognitive domains (episodic memory, speed-attention, visuospatial abilities, numeric reasoning, verbal fluency). Loneliness was also associated with informant-rated cognitive decline. The observed associations were significant controlling for depressive symptoms and living alone. The findings were generally consistent when loneliness was measured with 1-item or 3-item UCLA scale, were similar in subgroups that scored in the range of cognitive impairment and did not differ across age, sex, and other sociodemographic groups. Furthermore, the harmonized approach revealed replicable associations across samples from different world regions, including the United States, England, India, China, South Africa, Mexico, and Chile.

Previous research has found a negative association between loneliness and overall cognitive function (Harrington et al., 2023). The association between loneliness and other cognitive domains has been inconsistent, with studies reporting negative associations (Desai et al., 2023; Estrella et al., 2021) and others no associations (Kyröläinen & Kuperman, 2021; Windsor et al., 2020). The present meta-analysis found that loneliness was associated with worse performance in every domain measured, as well as overall cognitive function. For certain domains, like speed-attention, the association was significant (p < .01) and in the same direction in every sample that assessed the domain. The meta-analysis noted high heterogeneity for all domains assessed (except numeric reasoning), indicating that the effect sizes varied across samples, possibly due to sample composition, language, socioeconomic, or cultural factors. For instance, the samples from India, China, and South Africa included participants with lower education levels as compared to the samples from the United States and England. In addition to education, there might be other social and cultural factors not assessed in the current work that explains the heterogeneity. There are often concerns that patterns of associations found in the United States or other wealthy countries might not generalize to samples from countries with fewer economic resources (e.g., Smith et al., 2021). In such context, it was surprising to observe that loneliness had the weakest association with MMSE in the sample from the United States, but the effect size was stronger than average in the sample from England. Even with potential socio-cultural differences in loneliness (Akhter-Khan et al., 2024), its association with cognition function is broadly generalizable. These findings underscore the universality of loneliness and its harmful association with cognitive health.

Importantly, the current study links loneliness to cognitive function observed by a knowledgeable informant. That is, participants who reported higher levels of loneliness not only performed worse on the cognitive tasks, but also functioned worse in daily life, as observed by their informant. Informant ratings of cognitive function are critical to the diagnosis of dementia (American Psychiatric Association, 2013). Such ratings provide information about cognitive changes in everyday life that may not be detected on standardized cognitive tests and that might be noticeable only by close others (Pérez-Blanco et al., 2022).

Loneliness may affect cognition in multiple ways (Cacioppo & Hawkley, 2009). For instance, feeling lonely may reduce engagement in social activities, with a consequent reduction of cognitive stimulation, leaving individuals who experience loneliness more vulnerable to cognitive decline (Cacioppo & Hawkley, 2009; Kim et al., 2020). Further, loneliness is associated with depressive symptomatology (Cacioppo et al., 2010), unhealthy behaviors (e.g., physical inactivity), heightened stress, and poor sleep (Christiansen et al., 2016; Hawkley, Thisted, & Cacioppo, 2009), which may impair cognition over time (Dabiri, Mwendwa, & Campbell, 2024; Kim et al., 2020). Feeling lonely, however, is not the same as being alone and, more importantly, cannot be considered as a simple symptom of depression. Loneliness, for example, has an independent relation with cognitive impairment controlling for depressive symptoms, social isolation (e.g., participation in social activities) and health-related limitations (Luchetti et al., 2020). In the current analysis, the associations held controlling for depression and indices of isolation (i.e., living alone). Unfortunately, a harmonized measure of social participation/contact across the selected studies was not possible.

There was little evidence of moderation by depression or cognitive impairment. For speed-attention and verbal fluency, the association with loneliness was observed among individuals without depression, and for memory, among individuals without cognitive impairment. This result suggests that feelings of loneliness might be detrimental to cognition, particularly before the onset of clinical symptoms that characterize severe impairment. There was also limited evidence of moderation for most sociodemographic factors suggesting generalizability of the negative association between loneliness and cognitive-related outcomes across sociodemographic groups. In the samples from India, China, and Mexico, the negative association of loneliness with performance on the MMSE was stronger among individuals with lower education, who generally tend to be at greater risk for poor cognitive outcomes. Still, the observed moderations should be interpreted cautiously because interactions are difficult to replicate. For example, the meta-analysis pointed to a loneliness × sex interaction for informant-rated cognitive decline, but the interaction term was significant only in the Chilean sample and not in the other samples.

This study has several strengths, including the coordinated analysis and meta-analytic synthesis of the association between loneliness and harmonized cognitive measures across seven studies around the world. There were, however, limitations. First, this study is cross-sectional and does not consider the dynamic nature of loneliness. The frequency and intensity of loneliness may change over time (Hawkley & Kocherginsky, 2018) and have differential consequences for cognitive health (Li et al., 2022; Zhong, Chen, & Conwell, 2016). Moreover, there might be bidirectional associations between loneliness and cognitive function, with loneliness predicting cognitive decline and poor cognitive performance predicting increases in loneliness (Cachón-Alonso et al., 2023; Yin, Lassale, Steptoe, & Cadar, 2019; Zhong, Chen, Tu, & Conwell, 2017). Second, while the current analysis indicates loneliness is an important predictor of cognition independent of the measure used (1-item versus 3-item scale), some evidence suggests the association may vary based on loneliness scale and content of the items (Camacho et al., 2024; Luchetti et al., 2024; Shibata et al., 2021). More work is needed to understand how loneliness, including its temporal (transient versus persistent) and emotional and social facets, relate to cognition and specific cognitive domains. Third, our analysis accounted for possible mediators of the association between loneliness and cognition, such as depression (Dabiri et al., 2024). Future work could consider additional social and health-related covariates (e.g., social participation/contact; Luchetti et al., 2020). Fourth, this study used the MMSE to identify participants with cognitive impairment. MMSE, however, might not identify early stages of impairment (Salis, Costaggiu, & Mandas, 2023) and future work should investigate whether cognitive status moderates the association between loneliness and cognitive domains using a clinical classification of impairment or dementia. Lastly, the current work is one of the few studies that examined loneliness and cognition in samples from different world regions. Even though the observed associations did not depend on a specific country or region, it remains important to examine loneliness and cognitive health among other low- and middle-income countries, which have greater numbers of individuals living with dementia than high-income countries (Guerchet, Prince, & Prina, 2020).

In summary, the present research supports the literature that links loneliness and risk of dementia (Luchetti et al., 2024). Interventions that address loneliness might be effective in supporting cognition, particularly before the onset of clinical symptoms that characterize severe impairment (Joshi et al., 2024). While interventions might need cultural adaptations, the findings of this study suggest that the association between loneliness and cognitive function is not limited to the Western cultural context; it is evident across samples from diverse world regions.

## Supporting information

Lee et al. supplementary material Lee et al. supplementary material

## Supplementary material

The supplementary material for this article can be found at http://doi.org/10.1017/S003329172500011X.
