# Supplementary material for: Loneliness and cognition in older adults: A meta-analysis of harmonized studies from the United States, England, India, China, South Africa, Mexico, and Chile
Source: Psychol Med. 2025 Feb 20;55:e58. doi: 10.1017/S003329172500011X (PMC11939032; doi:10.1017/S003329172500011X)
Supplement: Lee et al. supplementary material [file S003329172500011Xsup001.docx]

Supplementary Material

**Loneliness and cognition in older adults: A meta-analysis of harmonized studies from the United States, England, India, China, South Africa, Mexico, and Chile**

Table S1

*Cognitive measures across studies*

| Domain | Measures | Test Description / Score | HRS | ELSA | LASI-DAD | CHARLS | DS-HAALSI | Mex-Cog | Chile-Cog |
| --- | --- | --- | --- | --- | --- | --- | --- | --- | --- |
| Global cognition | **Mini-Mental State Examination (MMSE)** | Max score 30 or 28; participants with more than 5 missing MMSE domains or tasks (Godin et al. 2017) were excluded from MMSE computation. | Max 3. Items = orientation time (5), orientation place (5), immediate and delayed word recall (3 + 3), spelling task (5), object naming (2), sentence (1), read and follow (1), 3-stage task (3), writing (1) and drawing (1). | Max 3. Note: Serial 7 (5) instead of the spelling task. | Max 3. Same as HRS. | Max 3. Note: Serial 7 (5) instead of the spelling task. | Max 3. Note: Serial 7 or spelling task (5). | Max 28. Note: Orientation place (3). | Max 3. Note: Digit backward or Serial 7 (5). |
| Episodic Memory | **CERAD Word List Memory** | Learning and Immediate Recall of 10 words (3 trials), score 0-3. | Similar across studies. | |  |  |  |  |  |
|  |  | Delayed Recall (1 trail), score 0-1. | Similar across studies. | |  |  |  |  |  |
|  |  | Recognition (20 words), score 0-2. | Similar across studies. | |  |  |  |  |  |
|  | **Logical Memory, Long Story.** Most studies used an adapted version of the Wechsler Memory Scale (‘Anna’ story). | Immediate Recall. Participants were given credit either for exact answers and/or approximate answers (2 or 1 point). | Exact answers only (25 items). Total score 0-25. | Same as HRS. | Same as HRS. | NA | Exact answers only (24 items). Total score 0-24. | Same as LASI-DAD. | Same as LASI-DAD. |
|  |  | Delayed Recall. Participants were given credit either for exact answers and/or approximate answers (2 or 1 points). | Exact answers only (25 items). Total score 0-25. | Same as HRS. | Same as HRS. | NA | Exact answers only (24 items). Total score 0-24. | Same as LASI-DAD. | Same as LASI-DAD. |
|  |  | Recognition. One point for each correct answer. | Score 0-15. | Score 0-15. | Score 0-15. | NA | Score 0-15. | NA | NA |
|  | **Logical Memory, Short Story.** Most studies used a version of a fire/assault (‘Brave Man’ story). | Immediate Recall. Participants were given credit either for exact answers and/or approximate answers (2 or 1 points). | Exact and approximate answers (6 items). Total score is 0-12. | Exact answers only (6 items). Total score is 0-6. | Exact and approximate answers (10 items). Score provided is comparable to HRS. | NA | Exact answers only (10 items). Total score is 0-1. | Same as HRS. | Same as HRS. |
|  |  | Delayed Recall. Participants were given credit either for exact answers and/or approximate answers (2 or 1 points). | Exact and approximate answers (6 items). Total score is 0-12. | Exact answers only (6 items). Total score is 0-6. | Exact and approximate answers (10 items). Score provided is comparable to HRS. | NA | Exact answers only (10 items). Total score is 0-1. | Same as HRS. | Same as HRS. |
| Speed-Attention | **Letter or Symbol Cancellation** | Point for each marked symbol/letter. The score is derived from how many letters/symbols they mark given a time. | Letter | Letter | Symbol | NA | Symbol | Symbol | Symbol |
|  | **Backward counting or naming** | Count backward from 100 or 2. Alternatively, naming backward (e.g., weekdays). | Counting (100). Number of correct counts. Time = 30 seconds. | Same as HRS. | NA | NA | Naming (days of the week). Total score 0-7. | Counting (20). Time = 60 seconds. The score ranged from 0-4.^a^ A second trial was offered. | Similar to HRS. Time = 90 seconds. |
|  | **Symbol Digit Modality Test** | Nine digits are presented associated with a symbol. The respondent has to fill in blank boxes with the symbol that correspond to each digit, according to the example given. | Time = 90 seconds. Score is the number of attempted pairings minus the number of mistakes or skipped pairings. | Same as HRS. | NA | NA | NA | Max score 56 (56 boxes). | Max score 56 (56 boxes). |
|  | **Trail Making A & B** | Trail A involves numbered circles and the respondent is asked to draw a line linking the circles in numeric order (1, 2, 3, etc.). Trail B involves numbered circles and circles containing letters, and the respondent is asked to draw a line linking the numbers and letters alternately (1, A, 2, B, 3, C, etc.). The task is timed. | The score is the time taken to complete each task. | Same as HRS. | NA | NA | NA | NA | NA |
| Visuospatial Ability | **Constructional Praxis** | Four sheets are typically presented: circle, rhombus, rectangle, and cube. The respondent has to copy the corresponding figures. In the recall task, is asked to draw from memory the four figures. | Max score 11 for immediate and delayed recall. | Same as HRS. | Same as HRS. | NA | Same as HRS. | NA | NA |
|  | **Raven's Matrixes** | Respondent is asked to identify the missing element that completes a pattern. Respondent is given 1 point for each pattern correctly identified. | Max score 17. | Same as HRS. | Same as HRS. | NA | Same as HRS. | NA | NA |
| Numeric Reasoning | **Number Series** | Respondent is presented with a series of numbers with one or two numbers missing. Participants are asked to identify the missing numbers. The test consists of a 6-item block adapted based on participants' performance. | Score based on HRS syntax. | | NA | NA | NA | NA | NA |
| Language | **Verbal Fluency** | Animal naming. The score reflected the total number of correct animals named om 1 minute. | Similar across studies. | |  |  |  |  |  |

*Note.* Studies are the Health and Retirement Study (HRS), the English Longitudinal Study of Ageing (ELSA), the Diagnostic Assessment of Dementia for the Longitudinal Aging Study in India (LASI-DAD), China Health and Retirement Longitudinal Study (CHARLS), Dementia Study of the Health and Aging in Africa (DS-HAALSI), the Mexican Cognitive Aging Ancillary Study (Mex-Cog) of the Mexican Health and Aging Study, and the Chile Cognitive Aging Study (Chile-Cog). NA = Not assessed. ^a^ The score was 4 = correct and within 10 seconds, 3 = correct within 11-20 seconds, 2 = correct within 21-30 seconds, 1 = correct within 31-59 seconds, 0 = incorrect and/or out of time.

Table S2

*Informant ratings across studies*

| Measure | Test Description / Score | HRS | ELSA | LASI-DAD | CHARLS | DS-HAALSI | Mex-Cog | Chile-Cog | |
| --- | --- | --- | --- | --- | --- | --- | --- | --- | --- |
| IQ-CODE | Response scale from 1= much better to 5 = much worse. Mean across items (score range 1-5). | 16-item version | Same as HRS. | Same as HRS. | 26-item version | Same as HRS. | NA | NA | |
| CSI-D | The presence of certain symptoms or behaviors was scored as 1. The sum was taken across items. | 15-item version Responses were coded: Yes, or Sometimes (=1) and No (=0). Score range 0-15. | Same as HRS. | Same as HRS. | 6-item version Responses were yes (=1) or no (=0). Score range 0-6. | Same as HRS. | Administered 28 questions on cognitive and functional decline (long-form). Six items were administered to all participants. Responses were coded: Yes, always or sometimes (=1) or No (=0). Score range 0-6. | Administered 28 questions on cognitive and functional decline (long-form). Six items were administered to all participants. Responses were coded: Yes, always or sometimes (=1) or No (=0). Score range 0-6. | |
| Blessed Dementia Rating Scale – Part 1 | Items (e.g., “remembering a short list of items such as a shopping list”) were coded as 1 (Some loss or Severe loss) or 0 (No loss).The sum was taken across items. | 8-item version Score range 0-8. | Same as HRS. | Skipped for ~1,500 participants. Not used in the analysis. | NA | Same as HRS. | NA | NA | |
| 10/66 | Items (e.g., “Has there been a change in his/her ability to handle money? as 1 (Some difficulty or Cannot do) or 0 (No difficulty). The sum was taken across items. | 5-item version Score range 0-5. | Same as HRS. | Same as HRS. | NA | Same as HRS. | NA | NA | |
| Informant Information | Age of the informant was reported in years across studies. Sex was recoded as 0 = male and 1 = female. Education reported in years or highest level. Relationship with the respondent was recoded as 1 = spouse and 0 = other across studies. Living arrangements were recoded 1= living together with the respondent, 0 = not living together/other frequency of contact. Additional information on the caregiver role (yes/no) and years of knowing the respondents were unavailable in some studies and thus were not included as covariates in the analysis. | | | | | | | |  |

*Note.* Studies are the Health and Retirement Study (HRS), the English Longitudinal Study of Ageing (ELSA), the Diagnostic Assessment of Dementia for the Longitudinal Aging Study in India (LASI-DAD), China Health and Retirement Longitudinal Study (CHARLS), Dementia Study of the Health and Aging in Africa (DS-HAALSI), the Mexican Cognitive Aging Ancillary Study (Mex-Cog) of the Mexican Health and Aging Study, and the Chile Cognitive Aging Study (Chile-Cog). NA = Not assessed.

Table S3

*Covariates across studies*

| Variable | Study | Description |
| --- | --- | --- |
| Age | All studies | Reported in years. |
|  | * CHARLS | Computed from date of birth. Date of birth was reported according to lunar calendar. We computed age from dates converted to solar calendar provided by the g2aging harmonized files. |
| Sex | All studies | Recoded 1 = female, 0 = male. |
| Race | HRS, ELSA | Recoded as 1 = Black/other or non-white, 0 = white. |
| Ethnicity | HRS | Recoded as 1= Hispanic/Latinx, 0 = Non-Hispanic/Latinx. |
| Education | HRS, Mex-Cog | Reported in years. |
|  | ELSA | Completed level of education. The original 7-point ordinal scale was: No qualification; foreign qualification or other; nvq1/gse other grade equivalent; nvq2/gce o level equivalent; nvq3/gce a level equivalent; higher ed below degree; nvq4/nvq5/degree or equivalent. |
|  | LASI-DAD | Completed level of education. Responses were harmonized by g2aging (10-point scale): Never attended school; less than primary (standard1-4); primary (standard 5-7); middle (standard 8-9); secondary (standard 10-11); higher secondary (standard12); diploma and certificate; graduate degree; post-graduate degree; professional course/degree. |
|  | CHARLS | Completed level of education. The original 11-point ordinal scale was recorded in 4 categories: No formal education/no finished primary education; elementary/middle school; high school or vocational school; degree or higher. |
|  | DS-HAALSI | Completed level of education. The ordinal scale included the following categories: No formal education; some primary (1-7 years); some secondary (8-11 years); secondary or more (12+ years). |
|  | Chile-Cog | Not retrieved. |
| Marital Status | All studies | Recoded 1 = married or cohabitation with a partner, 0 = single, separated, divorced, widowed. |
|  | * Chile-Cog | Not retrieved. |
| Depression | HRS, ELSA | CESD (8-item version), responses were yes (1) or no (0). The sum was taken across the time (range 0-7); the score excluded the item on loneliness. |
|  | LASI-DAD, CHARLS | CESD (10-item version), response scale 1-4. The mean was taken across items; the score excluded the item on loneliness. |
|  | DS-HAALSI | CESD (20-item version), response scale 0-3. The mean was taken across items; the score excluded the item on loneliness. |
|  | Mex-Cog | CESD (9-item version), responses were yes (1) or no (0). The sum was taken across the time (range 0-8); the score excluded the item on loneliness. |
|  | Chile-Cog | CESD (15-items), responses were yes (1) or no (0). The sum was taken across the time (range 0-15). The scale did not include items on loneliness. |
| Living Arrangements | All studies | No. of people in the household. Recoded 1 = living alone, 0 = living with others. |
|  | * Chile-Cog | Not retrieved. |
| Language | HRS | English or Spanish (coded as 1). Note: Results remained the same when including this covariate. |
|  | LASI-DAD | Hindi or other (coded as 1). Note: Results remained the same when including this covariate. |
|  | CHARLS | Mandarin Chinese or other (coded as 1); use of a translator (yes = 1). Note: Results remained the same when including these covariates. |
| Time | HRS, ELSA, | Time (years or months) between loneliness assessment and HCAP administration. Note: Results were the same when controlling for this covariate. |
|  | * Mex-Cog | Loneliness was derived from the same interview year of the HCAP assessment; information on dates of assessment was not available and thus we did not control for time (days/months) between regular and HCAP interviews. |
| *Note.* Studies are the Health and Retirement Study (HRS), the English Longitudinal Study of Ageing (ELSA), the Diagnostic Assessment of Dementia for the Longitudinal Aging Study in India (LASI-DAD), China Health and Retirement Longitudinal Study (CHARLS), Dementia Study of the Health and Aging in Africa (DS-HAALSI), the Mexican Cognitive Aging Ancillary Study (Mex-Cog) of the Mexican Health and Aging Study, and the Chile Cognitive Aging Study (Chile-Cog). Information on covariates was retrieved from regular interviews or HCAP. | | |

Table S4

*Descriptive statistics and zero-order correlations for each sample*

| Study: HRS | M (SD); range | 1 | 2 | 3 | 4 | 5 | 6 | 7 |
| --- | --- | --- | --- | --- | --- | --- | --- | --- |
| **1. Global cognition (MMSE)** | 27.11(3.24);1-30 | 1 | .621** | .651** | .586** | .416** | .452** | -.434** |
| **2. Episodic Memory (Composite)** | -0.01 (0.75); -3.67-1.96 | .621** | 1 | .584** | .557** | .383** | .540** | -.426** |
| *Word List* |  |  |  |  |  |  |  |  |
| Immediate Recall | 17.86 (4.87); 0-30 | .571** | .772** | .536** | .487** | .322** | .486** | -.389** |
| Delayed Recall | 5.31 (2.56); 0-10 | .531** | .785** | .506** | .469** | .305** | .475** | -.387** |
| Recognition | 18.70 (2.11); 0-20 | .504** | .665** | .398** | .380** | .165** | .346** | -.352** |
| *Short Story* |  |  |  |  |  |  |  |  |
| Immediate Recall | 7.27 (2.32);0-12 | .424** | .722** | .382** | .355** | .220** | .324** | -.280** |
| Delayed Recall | 5.21 (3.28); 0-12 | .365** | .752** | .367** | .380** | .263** | .371** | -.271** |
| *Long Story* |  |  |  |  |  |  |  |  |
| Immediate Recall | 10.18 (5.03);0-23 | .481** | .797** | .474** | .458** | .341** | .440** | -.291** |
| Delayed Recall | 7.59 (5.42);0-25 | .456** | .812** | .475** | .456** | .341** | .460** | -.297** |
| Recognition | 10.46 (2.65);0-15 | .412** | .675** | .389** | .366** | .250** | .333** | -.258** |
| **3. Speed–Attention (Composite)** | -0.04(0.87); -4.74-2.16 | .651** | .584** | 1 | .672** | .528** | .549** | -.445** |
| Letter/Symbol Cancellation | 15.22(5.05);0-37 | .441** | .417** | .805** | .444** | .267** | .384** | -.347** |
| Symbol Digit Modality Test | 33.59(12.04);0-71 | .558** | .560** | .899** | .638** | .500** | .507** | -.382** |
| Backward Counting/Naming | 30.22(10.88);0-70 | .530** | .441** | .770** | .511** | .464** | .453** | -.322** |
| Trails A | -53.64(36.74); -300--3 | .538** | .433** | .782** | .544** | .359** | .386** | -.336** |
| Trails B | -138.72(73.70); -300--32 | .494** | .459** | .767** | .556** | .489** | .460** | -.314** |
| **4. Visuospatial Ability (Composite)** | -0.01 (0.85); -3.69-1.24 | .586** | .557** | .672** | 1 | .565** | .516** | -.343** |
| Constructional Praxis | 8.37 (2.24);0-11 | .423** | .329** | .471** | .784** | .356** | .344** | -.224** |
| Constructional Praxis, Recall | 6.07 (3.14); 0-11 | .476** | .545** | .518** | .769** | .338** | .448** | -.355** |
| Raven Matrices | 12.73 (3.45); 0-17 | .530** | .492** | .635** | .903** | .571** | .466** | -.277** |
| **5. Numeric Reasoning** | 523.76 (30.67); 409-584 | .416** | .383** | .528** | .565** | 1 | .389** | -.186** |
| **6. Verbal Fluency** | 16.47 (6.41);0-43 | .452** | .540** | .549** | .516** | .389** | 1 | -.320** |
| **7. Informant Ratings (Composite)** | 0.02 (0.88); -2.00-3.36 | -.434** | -.426** | -.445** | -.343** | -.186** | -.320** | 1 |
| Jorm IQ-CODE | 3.06 (0.40);1-5 | -.253** | -.254** | -.241** | -.156** | -.023 | -.149** | .802** |
| CSI-D | 3.38 (3.02); 0-15 | -.415** | -.412** | -.402** | -.313** | -.191** | -.305** | .877** |
| Blessed Dementia Scale I | 2.14 (2.08); 0-8 | -.392** | -.386** | -.423** | -.334** | -.203** | -.294** | .904** |
| 10/66 | 1.01 (1.36); 0-5 | -.371** | -.360** | -.408** | -.323** | -.177** | -.298** | .871** |

*Note.* Values represent means (standard deviations) and ranges. Higher informant ratings indicate a higher decline.

** p <.01

| Study: ELSA | M (SD); range | 1 | 2 | 3 | 4 | 5 | 6 | 7 |
| --- | --- | --- | --- | --- | --- | --- | --- | --- |
| **1. Global cognition (MMSE)** | 26.43(3.32);9-30 | 1 | .630** | .623** | .577** | .489** | .421** | -.481** |
| **2. Episodic Memory (Composite)** | -0.02 (0.78); -2.89-1.56 | .630** | 1 | .592** | .561** | .438** | .457** | -.487** |
| *Word List* |  |  |  |  |  |  |  |  |
| Immediate Recall | 17.74 (5.28); 0-30 | .560** | .767** | .597** | .495** | .378** | .431** | -.492** |
| Delayed Recall | 5.08 (2.67); 0-10 | .534** | .795** | .561** | .490** | .360** | .442** | -.488** |
| Recognition | 18.32 (2.63); 0-20 | .453** | .679** | .437** | .431** | .323** | .342** | -.431** |
| *Short Story* |  |  |  |  |  |  |  |  |
| Immediate Recall | 4.02 (1.47); 0-6 | .484** | .774** | .406** | .408** | .317** | .315** | -.326** |
| Delayed Recall | 2.74 (1.96); 0-6 | .375** | .741** | .354** | .356** | .226** | .267** | -.273** |
| *Long Story* |  |  |  |  |  |  |  |  |
| Immediate Recall | 8.75 (4.70); 0-24 | .532** | .833** | .470** | .460** | .365** | .364** | -.379** |
| Delayed Recall | 6.63 (4.78); 0-21 | .487** | .836** | .460** | .448** | .355** | .341** | -.335** |
| Recognition | 11.13 (2.82); 0-15 | .412** | .691** | .342** | .355** | .311** | .290** | -.296** |
| **3. Speed–Attention (Composite)** | -0.05 (0.85); -3.68-2.17 | .623** | .592** | 1 | .627** | .542** | .507** | -.455** |
| Letter/Symbol Cancellation | 16.08(5.13); 0-35 | .391** | .435** | .795** | .421** | .315** | .363** | -.365** |
| Symbol Digit Modality Test | 32.99(12.66);0-72 | .515** | .536** | .878** | .574** | .512** | .431** | -.376** |
| Backward Counting/Naming | 30.60(11.10);0-74 | .522** | .419** | .763** | .486** | .474** | .408** | -.268** |
| Trails A | -57.40 (37.17); -300--4 | .448** | .415** | .756** | .520** | .375** | .336** | -.357** |
| Trails B | -123.33 (62.29); -300--10 | .469** | .459** | .750** | .417** | .419** | .344** | -.365** |
| **4. Visuospatial Ability (Composite)** | -0.03 (0.89); -4.49-1.02 | .577** | .561** | .627** | 1 | .593** | .434** | -.357** |
| Constructional Praxis | 9.48 (1.83); 0-11 | .405** | .345** | .446** | .787** | .415** | .283** | -.199** |
| Constructional Praxis, Recall | 7.54 (2.93); 0-11 | .450** | .530** | .490** | .791** | .425** | .366** | -.330** |
| Raven Matrices | 13.38 (3.53); 0-17 | .531** | .518** | .591** | .907** | .577** | .404** | -.324** |
| **5. Numeric Reasoning** | 527.38 (33.59); 409-584 | .489** | .438** | .542** | .593** | 1 | .356** | -.207** |
| **6. Verbal Fluency** | 18.31 (8.64); 0-51 | .421** | .457** | .507** | .434** | .356** | 1 | -.304** |
| **7. Informant Ratings (Composite)** | -0.00 (0.85); -4.16-2.94 | -.481** | -.487** | -.455** | -.357** | -.207** | -.304** | 1 |
| Jorm IQ-CODE | 3.17 (0.52); 1-5 | -.411** | -.373** | -.326** | -.281** | -.136** | -.209** | .773** |
| CSI-D | 3.54 (3.15); 0-15 | -.437** | -.457** | -.415** | -.307** | -.165** | -.289** | .878** |
| Blessed Dementia Scale I | 2.05 (2.02); 0-8 | -.455** | -.468** | -.453** | -.338** | -.223** | -.301** | .899** |
| 10/66 | 2.56 (0.92); 0-5 | -.335** | -.359** | -.348** | -.280** | -.183** | -.243** | .800** |

*Note.* Values represent means (standard deviations) and ranges. Higher informant ratings indicate a higher decline.

** p <.01

| Study: LASI-DAD | M (SD); range | 1 | 2 | 3 | 4 | 5 | 6 | 7 |
| --- | --- | --- | --- | --- | --- | --- | --- | --- |
| **1. Global cognition (MMSE)** | 22.79(5.29);0-30 | 1 | .637** | .547** | .630** | -- | .421** | -.426** |
| **2. Episodic Memory (Composite)** | -0.04 (0.76); -4.67-2.79 | .637** | 1 | .505** | .572** | -- | .424** | -.399** |
| *Word List* |  |  |  |  |  |  |  |  |
| Immediate Recall | 11.63 (5.03); 0-28 | .519** | .747** | .441** | .479** | -- | .407** | -.340** |
| Delayed Recall | 3.15 (2.30); 0-10 | .473** | .706** | .385** | .432** | -- | .359** | -.310** |
| Recognition | 16.15 (3.46); 0-20 | .546** | .659** | .386** | .479** | -- | .348** | -.297** |
| *Short Story* |  |  |  |  |  |  |  |  |
| Immediate Recall | 5.44 (3.05); 0-12 | .480** | .791** | .344** | .399** | -- | .330** | -.324** |
| Delayed Recall | 3.08 (3.50); 0-12 | .463** | .758** | .356** | .391** | -- | .271** | -.266** |
| *Long Story* |  |  |  |  |  |  |  |  |
| Immediate Recall | 3.98 (4.03); 0-24 | .455** | .785** | .370** | .403** | -- | .288** | -.278** |
| Delayed Recall | 2.94 (4.06); 0-25 | .464** | .788** | .404** | .434** | -- | .312** | -.286** |
| Recognition | 7.71 (3.00); 0-15 | .418** | .664** | .332** | .377** | -- | .226** | -.292** |
| **3. Speed–Attention (Composite)** | 0.00 (1.00); -0.84-5.93 | .547** | .505** | 1 | .623** | -- | .384** | -.296** |
| Letter/Symbol Cancellation | 7.09(8.41); 0-57 | .547** | .505** | 1 | .623** | -- | .384** | -.296** |
| Symbol Digit Modality Test | -- | -- | -- | -- | -- | -- | -- | -- |
| Backward Counting/Naming | -- | -- | -- | -- | -- | -- | -- | -- |
| Trails A | -- | -- | -- | -- | -- | -- | -- | -- |
| Trails B | -- | -- | -- | -- | -- | -- | -- | -- |
| **4. Visuospatial Ability (Composite)** | -0.04 (0.84); -2.39-2.62 | .630** | .572** | .623** | 1 | -- | .364** | -.352** |
| Constructional Praxis | 5.74 (3.20); 0-11 | .588** | .466** | .616** | .770** | -- | .324** | -.288** |
| Constructional Praxis, Recall | 2.80 (2.71); 0-11 | .474** | .478** | .542** | .764** | -- | .285** | -.261** |
| Raven Matrices | 7.68 (3.21); 0-17 | .515** | .471** | .457** | .882** | -- | .298** | -.296** |
| **5. Numeric Reasoning** | Not Assessed | -- | -- | -- | -- | -- | -- | -- |
| **6. Verbal Fluency** | 11.46 (4.53); 0-32 | .421** | .424** | .384** | .364** | -- | 1 | 1 |
| **7. Informant Ratings (Composite)** | 0.00 (0.84); -2.21-2.66 | -.426** | -.399** | -.296** | -.352** | -- | -.282** | 1.000** |
| Jorm IQ-CODE | 3.44 (0.54); 1-5 | -.400** | -.374** | -.297** | -.352** | -- | -.286** | .840** |
| CSI-D | 4.19 (3.50); 0-15 | -.338** | -.318** | -.203** | -.258** | -- | -.190** | .838** |
| Blessed Dementia Scale I | -- | -- | -- | -- | -- | -- | -- | -- |
| 10/66 | 1.74 (1.56); 0-5 | -.337** | -.314** | -.246** | -.276** | -- | -.233** | .847** |

*Note.* Values represent means (standard deviations) and ranges. Higher informant ratings indicate a higher decline.

** p <.01

| Study: CHARLS | M (SD); range | 1 | 2 | 3 | 4 | 5 | 6 | 7 |
| --- | --- | --- | --- | --- | --- | --- | --- | --- |
| **1. Global cognition (MMSE)** | 20.06(6.28);0-30 | 1 | .684** | -- | -- | -- | .458** | -.226** |
| **2. Episodic Memory (Composite)** | -0.07 (0.92); -2.68-2.38 | .684** | 1 | -- | -- | -- | .439** | -.183** |
| *Word List* |  |  |  |  |  |  |  |  |
| Immediate Recall | 11.55 (6.62); 0-30 | .641** | .916** | -- | -- | -- | .432** | -.163** |
| Delayed Recall | 3.46 (2.70); 0-10 | .589** | .894** | -- | -- | -- | .395** | -.191** |
| Recognition | 17.18 (3.43); 0-20 | .555** | .802** | -- | -- | -- | .293** | -.157** |
| *Short Story* |  |  |  |  |  |  |  |  |
| Immediate Recall | -- | -- | -- | -- | -- | -- | -- | -- |
| Delayed Recall | -- | -- | -- | -- | -- | -- | -- | -- |
| *Long Story* |  |  |  |  |  |  |  |  |
| Immediate Recall | -- | -- | -- | -- | -- | -- | -- | -- |
| Delayed Recall | -- | -- | -- | -- | -- | -- | -- | -- |
| Recognition | -- | -- | -- | -- | -- | -- | -- | -- |
| **3. Speed–Attention (Composite)** | Not Assessed | -- | -- | -- | -- | -- | -- | -- |
| **4. Visuospatial Ability (Composite)** | Not Assessed | -- | -- | -- | -- | -- | -- | -- |
| **5. Numeric Reasoning** | Not Assessed | -- | -- | -- | -- | -- | -- | -- |
| **6. Verbal Fluency** | 11.82 (4.90); 0-71 | .458** | .439** | -- | -- | -- | 1 | -.137** |
| **7. Informant Ratings (Composite)** | 0.00 (0.92); -2.64-2.41 | -.226** | -.183** | -- | -- | -- | -.137** | 1 |
| Jorm IQ-CODE | 3.47 (0.59); 1-5 | -.215** | -.173** | -- | -- | -- | -.127** | .920** |
| CSI-D | 1.99 (1.80); 0-6 | -.203** | -.166** | -- | -- | -- | -.126** | .920** |
| Blessed Dementia Scale I | -- | -- | -- | -- | -- | -- | -- | -- |
| 10/66 | -- | -- | -- | -- | -- | -- | -- | -- |

*Note.* Values represent means (standard deviations) and ranges. Higher informant ratings indicate a higher decline.

** p <.01

| Study: DS-HAALSI | M (SD); range | 1 | 2 | 3 | 4 | 5 | 6 | 7 |
| --- | --- | --- | --- | --- | --- | --- | --- | --- |
| **1. Global cognition (MMSE)** | 18.59(5.11);5-29 | 1.000** | .649** | .647** | .566** | -- | .333** | -.581** |
| **2. Episodic Memory (Composite)** | -0.01 (0.76); -2.59-1.80 | .649** | 1.000** | .558** | .524** | -- | .354** | -.515** |
| *Word List* |  |  |  |  |  |  |  |  |
| Immediate Recall | 12.15 (4.44); 0-24 | .616** | .735** | .460** | .465** | -- | .248** | -.443** |
| Delayed Recall | 3.71 (1.98); 0-10 | .565** | .701** | .433** | .470** | -- | .186** | -.406** |
| Recognition | 15.27 (3.03); 0-20 | .521** | .663** | .472** | .449** | -- | .293** | -.444** |
| *Short Story* |  |  |  |  |  |  |  |  |
| Immediate Recall | 5.83 (2.29); 0-10 | .504** | .855** | .435** | .365** | -- | .351** | -.459** |
| Delayed Recall | 4.88 (2.52); 0-10 | .435** | .836** | .425** | .338** | -- | .286** | -.357** |
| *Long Story* |  |  |  |  |  |  |  |  |
| Immediate Recall | 8.54 (4.18); 0-24 | .505** | .815** | .418** | .399** | -- | .278** | -.358** |
| Delayed Recall | 6.98 (4.21); 0-18 | .444** | .811** | .409** | .388** | -- | .208** | -.302** |
| Recognition | 8.96 (1.92); 0-14 | .300** | .526** | .316** | .300** | -- | .222** | -.303** |
| **3. Speed–Attention (Composite)** | -0.04 (0.88); -1.94-1.98 | .647** | .558** | 1.000** | .559** | -- | .338** | -.443** |
| Letter/Symbol Cancellation | 15.55(12.33);0-54 | .443** | .512** | .840** | .460** | -- | .279** | -.322** |
| Symbol Digit Modality Test | -- | -- | -- | -- | -- | -- | -- | -- |
| Backward Counting/Naming | 4.89(2.53);0-7 | .650** | .462** | .867** | .494** | -- | .279** | -.422** |
| Trails A | -- | -- | -- | -- | -- | -- | -- | -- |
| Trails B | -- | -- | -- | -- | -- | -- | -- | -- |
| **4. Visuospatial Ability (Composite)** | -0.02 (0.86); -2.63-2.27 | .566** | .524** | .559** | 1.000** | -- | .188** | -.447** |
| Constructional Praxis | 5.26 (2.57); 0-11 | .538** | .409** | .577** | .727** | -- | .242** | -.342** |
| Constructional Praxis, Recall | 2.94 (2.69); 0-11 | .482** | .435** | .426** | .759** | -- | .130** | -.264** |
| Raven Matrices | 7.39 (2.81); 0-16 | .463** | .443** | .464** | .901** | -- | .126** | -.400** |
| **5. Numeric Reasoning** | Not Assessed | -- | -- | -- | -- | -- | -- | -- |
| **6. Verbal Fluency** | 10.61 (3.67); 0-25 | .333** | .354** | .338** | .188** | -- | 1.000** | -.315** |
| **7. Informant Ratings (Composite)** | 0.00 (0.83); -0.86-3.34 | -.581** | -.515** | -.443** | -.447** | -- | -.315** | 1.000** |
| Jorm IQ-CODE | 3.30 (0.43); 2-5 | -.558** | -.533** | -.473** | -.395** | -- | -.227** | .821** |
| CSI-D | 2.90 (3.40); 0-15 | -.473** | -.404** | -.361** | -.385** | -- | -.280** | .797** |
| Blessed Dementia Scale I | 1.34 (2.32); 0-8 | -.428** | -.361** | -.315** | -.315** | -- | -.255** | .825** |
| 10/66 | 0.92 (1.23); 0-5 | -.456** | -.414** | -.329** | -.367** | -- | -.280** | .874** |

*Note.* Values represent means (standard deviations) and ranges. Higher informant ratings indicate a higher decline.

** p <.01

| Study: Mex-Cog | M (SD); range | 1 | 2 | 3 | 4 | 5 | 6 | 7 |
| --- | --- | --- | --- | --- | --- | --- | --- | --- |
| **1. Global cognition (MMSE)** | 20.09(4.17);2-28 | 1 | .618** | .691** | .576** | -- | .512** | -.314** |
| **2. Episodic Memory (Composite)** | -0.05 (0.79); -2.93-2.59 | .618** | 1 | .559** | .432** | -- | .492** | -.273** |
| *Word List* |  |  |  |  |  |  |  |  |
| Immediate Recall | 12.19 (4.16); 0-30 | .499** | .718** | .515** | .384** | -- | .449** | -.248** |
| Delayed Recall | 3.00 (2.10); 0-10 | .443** | .709** | .410** | .335** | -- | .380** | -.254** |
| Recognition | 16.52 (2.58); 8-20 | .458** | .671** | .430** | .340** | -- | .352** | -.220** |
| *Short Story* |  |  |  |  |  |  |  |  |
| Immediate Recall | 5.72 (2.43); 0-12 | .412** | .770** | .371** | .279** | -- | .313** | -.164** |
| Delayed Recall | 4.50 (2.61); 0-12 | .396** | .761** | .336** | .298** | -- | .289** | -.154** |
| *Long Story* |  |  |  |  |  |  |  |  |
| Immediate Recall | 11.70 (7.61); 0-42 | .491** | .833** | .443** | .343** | -- | .374** | -.188** |
| Delayed Recall | 9.38 (7.49); 0-49 | .480** | .837** | .421** | .344** | -- | .358** | -.176** |
| Recognition | -- | -- | -- | -- | -- | -- | -- | -- |
| **3. Speed–Attention (Composite)** | -0.05 (0.87); -2.05-2.15 | .691** | .559** | 1 | .612** | -- | .563** | -.269** |
| Letter/Symbol Cancellation | 23.90(14.77);0-60 | .566** | .508** | .862** | .562** | -- | .507** | -.245** |
| Symbol Digit Modality Test | 18.60(12.13);0-56 | .580** | .511** | .878** | .573** | -- | .504** | -.203** |
| Backward Counting/Naming | 2.75(1.34);0-4 | .575** | .365** | .793** | .425** | -- | .392** | -.184** |
| Trails A | -- | -- | -- | -- | -- | -- | -- | -- |
| Trails B | -- | -- | -- | -- | -- | -- | -- | -- |
| **4. Visuospatial Ability (Composite)** | -0.04 (0.93); -3.00-1.43 | .576** | .432** | .612** | 1 | -- | .401** | -.200** |
| Constructional Praxis | 7.98 (2.65); 0-11 | .542** | .350** | .567** | .901** | -- | .345** | -.178** |
| Constructional Praxis, Recall | 5.48 (3.21); 0-11 | .482** | .440** | .550** | .893** | -- | .383** | -.178** |
| Raven Matrices | -- | -- | -- | -- | -- | -- | -- | -- |
| **5. Numeric Reasoning** | Not Assessed | -- | -- | -- | -- | -- | -- | -- |
| **6. Verbal Fluency** | 13.83 (5.04); 0-33 | .512** | .492** | .563** | .401** | -- | 1 | -.224** |
| **7. Informant Ratings (Composite)** | 0.00 (1.00); -0.78-3.91 | -.314** | -.273** | -.269** | -.200** | -- | -.224** | 1 |
| Jorm IQ-CODE | -- | -- | -- | -- | -- | -- | -- | -- |
| CSI-D | 1.00 (1.28); 0-6 | -.314** | -.273** | -.269** | -.200** | -- | -.224** | 1 |
| Blessed Dementia Scale I | -- | -- | -- | -- | -- | -- | -- | -- |
| 10/66 | -- | -- | -- | -- | -- | -- | -- | -- |

*Note.* Values represent means (standard deviations) and ranges. Higher informant ratings indicate a higher decline.

** p <.01

| Study: Chile-Cog | M (SD); range | 1 | 2 | 3 | 4 | 5 | 6 | 7 |
| --- | --- | --- | --- | --- | --- | --- | --- | --- |
| **1. Global cognition (MMSE)** | 25.75(3.74);12-30 | 1 | .575** | .572** | .435** | -- | .438** | -.388** |
| **2. Episodic Memory (Composite)** | -0.03 (0.81); -3.85-2.44 | .575** | 1 | .566** | .448** | -- | .501** | -.366** |
| *Word List* |  |  |  |  |  |  |  |  |
| Immediate Recall | 13.60 (4.32); 1-30 | .442** | .731** | .476** | .371** | -- | .403** | -.290** |
| Delayed Recall | 4.14 (1.90); 1-10 | .268** | .664** | .351** | .300** | -- | .286** | -.182** |
| Recognition | 15.95 (2.93); 0-20 | .458** | .668** | .392** | .293** | -- | .331** | -.316** |
| *Short Story* |  |  |  |  |  |  |  |  |
| Immediate Recall | 7.10 (2.65); 0-12 | .419** | .780** | .368** | .268** | -- | .348** | -.234** |
| Delayed Recall | 5.62 (3.03); 0-12 | .435** | .789** | .393** | .320** | -- | .373** | -.264** |
| *Long Story* |  |  |  |  |  |  |  |  |
| Immediate Recall | 17.59 (9.19); 0-50 | .472** | .850** | .496** | .388** | -- | .431** | -.272** |
| Delayed Recall | 14.30 (9.65); 0-45 | .450** | .847** | .530** | .424** | -- | .444** | -.255** |
| Recognition | -- | -- | -- | -- | -- | -- | -- | -- |
| **3. Speed–Attention (Composite)** | -0.04 (0.88);-1.96-2.55 | .572** | .566** | 1 | .672** | -- | .589** | -.324** |
| Letter/Symbol Cancellation | 27.98(15.06);0-60 | .512** | .493** | .879** | .588** | -- | .518** | -.263** |
| Symbol Digit Modality Test | 25.69(14.34);0-56 | .480** | .481** | .884** | .594** | -- | .528** | -.287** |
| Backward Counting/Naming | 21.18(10.29);0-64 | .485** | .474** | .837** | .544** | -- | .467** | -.251** |
| Trails A | -- | -- | -- | -- | -- | -- | -- | -- |
| Trails B | -- | -- | -- | -- | -- | -- | -- | -- |
| **4. Visuospatial Ability (Composite)** | -0.04 (0.89); -3.00-1.67 | .435** | .448** | .672** | 1 | -- | .448** | -.231** |
| Constructional Praxis | 7.96 (2.32); 1-11 | .413** | .356** | .593** | .885** | -- | .377** | -.188** |
| Constructional Praxis, Recall | 4.59 (3.18); 0-11 | .331** | .417** | .582** | .880** | -- | .397** | -.187** |
| Raven Matrices | -- | -- | -- | -- | -- | -- | -- | -- |
| **5. Numeric Reasoning** | Not Assessed | -- | -- | -- | -- | -- | -- | -- |
| **6. Verbal Fluency** | 15.08 (5.26); 2-36 | .438** | .501** | .589** | .448** | -- | 1 | -.283** |
| **7. Informant Ratings (Composite)** | 0.00 (1.00); -0.70-3.71 | -.388** | -.366** | -.324** | -.231** | -- | -.283** | 1 |
| Jorm IQ-CODE | -- | -- | -- | -- | -- | -- | -- | -- |
| CSI-D | 0.95 (1.36); 0-6 | -.388** | -.366** | -.324** | -.231** | -- | -.283** | 1 |
| Blessed Dementia Scale I | -- | -- | -- | -- | -- | -- | -- | -- |
| 10/66 | -- | -- | -- | -- | -- | -- | -- | -- |

*Note.* Values represent means (standard deviations) and ranges. Higher informant ratings indicate a higher decline.

** p <.01

Table S5

*Associations with overall cognition and informant-rated cognitive decline controlling for depressive symptoms*

| **Overall Cognition** | | Regressions | | Meta-analysis | |
| --- | --- | --- | --- | --- | --- |
| Studies (7) |  | β | p | Fisher’s z (95% CI) | Weight |
| HRS |  | -.030 | .098 | -.030 (-.067, .007) | 12.15 |
| ELSA |  | -.122 | <.001 | -.123 (-.183, -.062) | 4.56 |
| LASI-DAD |  | -.027 | .058 | -.027 (-.058, .004) | 17.18 |
| CHARLS |  | -.036 | <.001 | -.036 (-.056, -.016) | 41.23 |
| DS-HAALSI |  | -.020 | .583 | -.020 (-.098, .058) | 2.68 |
| Mex-Cog |  | -.045 | .005 | -.045 (-.080, -.010) | 13.74 |
| Chile-Cog |  | -.045 | .067 | -.045 (-.089, -.001) | 8.45 |
| Overall |  | Estimate = -.039 (-.052, -.026), p <.001 | | | |
| Heterogeneity | | Q = 8.72, I^2^ (%) = 0.00, tau^2^ = .000 | | | |
| **Informant-rated cognitive decline** | | Regressions | | Meta-analysis | |
| Studies (7) |  | β | p | Fisher’s z (95% CI) | Weight |
| HRS |  | .102 | <.001 | .102 (.064, .141) | 15.28 |
| ELSA |  | .099 | .005 | .099 (.034, .165) | 11.39 |
| LASI-DAD |  | .022 | .169 | .022 (-.009, .053) | 16.31 |
| CHARLS |  | .018 | .136 | .018 (-.003, .039) | 17.54 |
| DS-HAALSI |  | .012 | .770 | .012 (-.067, .091) | 9.71 |
| Mex-Cog |  | .116 | <.001 | .117 (.082, .151) | 15.81 |
| Chile-Cog |  | .096 | .001 | .096 (.049, .144) | 13.98 |
| Overall |  | Estimate = .067(.031,.102), p <.001 | | | |
| Heterogeneity | | Q = 39.88, I^2^ (%) = 82.72, tau^2^ = .001 | | | |

*Note.* Studies are the Health and Retirement Study (HRS), the English Longitudinal Study of Ageing (ELSA), the Diagnostic Assessment of Dementia for the Longitudinal Aging Study in India (LASI-DAD), China Health and Retirement Longitudinal Study (CHARLS), Dementia Study of the Health and Aging in Africa (DS-HAALSI), the Mexican Cognitive Aging Ancillary Study (Mex-Cog) of the Mexican Health and Aging Study, and the Chile Cognitive Aging Study (Chile-Cog). βs are standardized coefficients from regressions within each sample; for informant ratings, higher scores indicate cognitive decline. We further report Fisher’s z (95% Confidence Intervals) and weights for each study included in the meta-analysis. Results controlling for age, sex, education, marital status, and race and ethnicity (where possible) are illustrated in Figures 1 and 2; the analysis reported here further accounted for depressive symptoms.

Table S6

*Associations with overall cognition and informant-rated cognitive decline controlling for living arrangements*

| **Overall Cognition** | | Regressions | | Meta-analysis | |
| --- | --- | --- | --- | --- | --- |
| Studies (7) |  | β | p | Fisher’s z (95% CI) | Weight |
| HRS |  | -0.048 | .005 | -.048 (-.085, -.011) | 17.93 |
| ELSA |  | -0.140 | <.001 | -.141 (-.201, -.081) | 10.99 |
| LASI-DAD |  | -0.085 | <.001 | -.085 (-.116, -.054) | 20.17 |
| CHARLS |  | -0.102 | <.001 | -.102 (-.122, -.082) | 24.50 |
| DS-HAALSI |  | -0.082 | .010 | -.082 (-.161, -.004) | 7.66 |
| Mex-Cog |  | -0.052 | <.001 | -.052 (-.087, -.017) | 18.75 |
| Chile-Cog |  | -- |  | -- | -- |
| Overall |  | Estimate = -.082 (-.108, -.057), p <.001 | | | |
| Heterogeneity | | Q = 13.53, I^2^ (%) = 63.84, tau^2^ = .001 | | | |
| **Informant-rated cognitive decline** | | Regressions | | Meta-analysis | |
| Studies (7) |  | β | p | Fisher’s z (95% CI) | Weight |
| HRS |  | 0.159 | <.001 | .160 (.122, .199) | 12.88 |
| ELSA |  | 0.157 | <.001 | .158 (.093, .224) | 4.48 |
| LASI-DAD |  | 0.157 | <.001 | .158 (.127, .189) | 19.74 |
| CHARLS |  | 0.150 | <.001 | .151 (.130, .172) | 44.02 |
| DS-HAALSI |  | 0.245 | <.001 | .250 (.171, .329) | 3.10 |
| Mex-Cog |  | 0.150 | <.001 | .151 (.116, .186) | 15.79 |
| Chile-Cog |  | -- |  | -- | -- |
| Overall |  | Estimate =.157 (.143,.171), p <.001 | | | |
| Heterogeneity | | Q = 5.84, I^2^ (%) = 0.01, tau^2^ = .000 | | | |

*Note.* Studies are the Health and Retirement Study (HRS), the English Longitudinal Study of Ageing (ELSA), the Diagnostic Assessment of Dementia for the Longitudinal Aging Study in India (LASI-DAD), China Health and Retirement Longitudinal Study (CHARLS), Dementia Study of the Health and Aging in Africa (DS-HAALSI), the Mexican Cognitive Aging Ancillary Study (Mex-Cog) of the Mexican Health and Aging Study. βs are standardized coefficients from regressions within each sample; for informant ratings, higher scores indicate cognitive decline. We further report Fisher’s z (95% Confidence Intervals) and weights for each study included in the meta-analysis. Results controlling for age, sex, education, marital status, and race and ethnicity (where possible) are illustrated in Figures 1 and 2; the analysis reported here further accounted for living arrangements (i.e., living alone). Information on living arrangements was not retrieved for the Chile Cognitive Aging Study (Chile-Cog) and thus the study was not included in this follow-up analysis.

Table S7

*Loneliness association with cognitive tasks and informant scales*

| **CERAD Word List Memory – Immediate Recall** | | Regressions | | | Meta-analysis | |
| --- | --- | --- | --- | --- | --- | --- |
| Studies (7) | N | β | | p | Fisher’s z (95% CI) | Weight |
| HRS | 2,814 | -.050 | | .004 | -.050 (-.087, -.013) | 15.58 |
| ELSA | 1,056 | -.080 | | .005 | -.080 (-.141, -.020) | 9.09 |
| LASI-DAD | 3,966 | -.065 | | <.001 | -.065 (-.096, -.034) | 17.79 |
| CHARLS | 8,442 | -.084 | | <.001 | -.084 (-.106, -.063) | 21.79 |
| DS-HAALSI | 630 | -.017 | | .636 | -.017 (-.095, .061) | 6.26 |
| Mex-Cog | 3,185 | -.034 | | .028 | -.034 (-.069, .001) | 16.39 |
| Chile-Cog | 1,953 | -.119 | | <.001 | -.120 (-.164, -.075) | 13.10 |
| Overall |  | Estimate = -.067 (-.090, -.045), p <.001 | | | | |
| Heterogeneity |  | | Q = 13.70, I^2^ (%) = 57.45, tau^2^ = .001 | | | |
| **CERAD Word List Memory – Delayed Recall** | | Regressions | | | Meta-analysis | |
| Studies (7) | N | β | | p | Fisher’s z (95% CI) | Weight |
| HRS | 2,820 | -.034 | | .045 | -.034 (-.071, .003) | 15.79 |
| ELSA | 1,059 | -.044 | | .112 | -.044 (-.104, .016) | 8.84 |
| LASI-DAD | 3,939 | -.057 | | <.001 | -.057 (-.088, -.026) | 18.23 |
| CHARLS | 8,008 | -.094 | | <.001 | -.094 (-.116, -.072) | 22.73 |
| DS-HAALSI | 630 | -.040 | | .273 | -.040 (-.118, .038) | 5.97 |
| Mex-Cog | 3,086 | -.041 | | .014 | -.041 (-.076, -.006) | 16.46 |
| Chile-Cog | 1,681 | -.085 | | <.001 | -.085 (-.133, -.037) | 11.97 |
| Overall |  | Estimate = -.060 (-.082,-.039), p <.001 | | | | |
| Heterogeneity |  | Q = 13.06, I^2^ (%) = 52.16, tau^2^ = .000 | | | | |
| **CERAD Word List Memory – Recognition** | | Regressions | | | Meta-analysis |  |
| Studies (7) | N | β | | p | Fisher’s z (95% CI) | Weight |
| HRS | 2,817 | -.017 | | .346 | -.017 (-.054, .020) | 15.59 |
| ELSA | 1,057 | -.028 | | .361 | -.028 (-.088,.032) | 10.80 |
| LASI-DAD | 3,894 | -.059 | | <.001 | -.059 (-.090,-.028) | 16.83 |
| CHARLS | 7,664 | -.094 | | <.001 | -.094 (-.117,-.072) | 18.74 |
| DS-HAALSI | 630 | -.109 | | .004 | -.109 (-.188,-.031) | 8.09 |
| Mex-Cog | 3,086 | -.027 | | .115 | -.027 (-.062,.008) | 15.96 |
| Chile-Cog | 1,967 | -.102 | | <.001 | -.102 (-.147,-.058) | 13.98 |
| Overall |  | Estimate = -.061 (-.089, -.032), p <.001 | | | | |
| Heterogeneity |  | Q = 23.05, I^2^ (%) = 72.98, tau^2^ = .001 | | | | |

(continues)

| **Logical Memory, Long Story – Immediate Recall** | | Regressions | | | Meta-analysis | |
| --- | --- | --- | --- | --- | --- | --- |
| Studies (6) | N | β | | p | Fisher’s z (95% CI) | Weight |
| HRS | 2,800 | -.032 | | .064 | -.032 (-.069, .005) | 18.32 |
| ELSA | 1,056 | -.063 | | .033 | -.063 (-.123, -.003) | 14.76 |
| LASI-DAD | 3,765 | -.039 | | .012 | -.039 (-.071, -.007) | 19.03 |
| CHARLS | 630 | -.006 | | .875 | -.006 (-.084, .072) | 12.18 |
| Mex-Cog | 3,007 | -.032 | | .061 | -.032 (-.068, .004) | 18.51 |
| Chile-Cog | 1,937 | -.153 | | <.001 | -.154 (-.199, -.110) | 17.20 |
| Overall |  | Estimate = -.056 (-.097, -.014), p = .008 | | | | |
| Heterogeneity |  | | Q = 24.59, I^2^ (%) = 81.39, tau^2^ = .002 | | | |
| **Logical Memory, Long Story – Delayed Recall** | | Regressions | | | Meta-analysis | |
| Studies (6) | N | β | | p | Fisher’s z (95% CI) | Weight |
| HRS | 2,808 | -.027 | | .123 | -.027 (-.064, .010) | 17.37 |
| ELSA | 1,024 | -.044 | | .140 | -.044 (-.105, .017) | 15.97 |
| LASI-DAD | 3,700 | -.051 | | .001 | -.051 (-.083, -.019) | 17.59 |
| DS-HAALSI | 619 | .117 | | .002 | .118 (.039, .197) | 14.74 |
| Mex-Cog | 2,841 | -.025 | | .160 | -.025 (-.062, .012) | 17.38 |
| Chile-Cog | 1,887 | -.166 | | <.001 | -.168 (-.213, -.122) | 16.96 |
| Overall |  | Estimate = -.036 (-.105,.033), p = .303 | | | | |
| Heterogeneity |  | Q = 46.59, I^2^ (%) = 93.19, tau^2^ = .007 | | | | |
| **Logical Memory, Long Story – Recognition** | | Regressions | | | Meta-analysis |  |
| Studies (4) | N | β | | p | Fisher’s z (95% CI) | Weight |
| HRS | 2,762 | -.028 | | .120 | -.028 (-.065, .009) | 34.11 |
| ELSA | 1,032 | -.057 | | .064 | -.057 (-.118, .004) | 12.73 |
| LASI-DAD | 3,676 | -.041 | | .009 | -.041 (-.073, -.009) | 45.39 |
| DS-HAALSI | 630 | -.069 | | .089 | -.069 (-.147, .009) | 7.76 |
| Overall |  | Estimate = -.041 (-.063, -.019), p <.001 | | | | |
| Heterogeneity |  | Q = 1.23, I^2^ (%) = .08, tau^2^ = .000 | | | | |
| **Logical Memory, Short Story – Immediate Recall** | | Regressions | | | Meta-analysis |  |
| Studies (6) | N | β | | p | Fisher’s z (95% CI) | Weight |
| HRS | 2,819 | -.034 | | .061 | -.034 (-.071, .003) | 19.06 |
| ELSA | 1,060 | -.056 | | .069 | -.056 (-.116, .004) | 13.57 |
| LASI-DAD | 3,842 | -.039 | | .012 | -.039 (-.071, -.007) | 20.38 |
| DS-HAALSI | 630 | -.088 | | .024 | -.088 (-.167, -.010) | 10.31 |
| Mex-Cog | 3,070 | -.011 | | .535 | -.011 (-.046, .024) | 19.45 |
| Chile-Cog | 1,967 | -.118 | | <.001 | -.119 (-.163, -.074) | 17.24 |
| Overall |  | Estimate = -.054 (-.086,-.021), p = .001 | | | | |
| Heterogeneity |  | Q = 15.90, I^2^ (%) = 70.03, tau^2^ = .001 | | | | |

(continues)

| **Logical Memory, Short Story – Delayed Recall** | | Regressions | | | Meta-analysis | |
| --- | --- | --- | --- | --- | --- | --- |
| Studies (6) | N | β | | p | Fisher’s z (95% CI) | Weight |
| HRS | 2,821 | -.028 | | .131 | -.028 (-.065, .009) | 18.78 |
| ELSA | 1,028 | -.002 | | .937 | -.002 (-.063, .059) | 14.05 |
| LASI-DAD | 3,815 | -.051 | | .001 | -.051 (-.083, -.019) | 19.77 |
| CHARLS | 619 | .007 | | .866 | .007 (-.072, .086) | 11.13 |
| Mex-Cog | 2,957 | -.016 | | .366 | -.016 (-.052, .020) | 18.94 |
| Chile-Cog | 1,967 | -.121 | | <.001 | -.122 (-.166, -.077) | 17.33 |
| Overall |  | Estimates = -.039 (-.075, -.002), p = .037 | | | | |
| Heterogeneity |  | | Q = 18.42, I^2^ (%) = 75.68, tau^2^ = .002 | | | |
| **Symbol or Letter Cancellation** | | Regressions | | | Meta-analysis | |
| Studies (6) | N | β | | p | Fisher’s z (95% CI) | Weight |
| HRS | 2,730 | -.078 | | <.001 | -.078 (-.116, -.041) | 18.77 |
| ELSA | 999 | -.121 | | <.001 | -.122 (-.184, -.059) | 14.38 |
| LASI-DAD | 3,905 | -.065 | | <.001 | -.065 (-.096, -.034) | 19.82 |
| DS-HAALSI | 533 | -.099 | | .011 | -.099 (-.184, -.014) | 10.85 |
| Mex-Cog | 3,035 | -.061 | | <.001 | -.061 (-.097, -.025) | 19.11 |
| Chile-Cog | 1,741 | -.180 | | <.001 | -.182 (-.229, -.135) | 17.06 |
| Overall |  | Estimate = -.099 (-.137, -.060), p <.001 | | | | |
| Heterogeneity |  | Q = 21.05, I^2^ (%) = 77.67, tau^2^ = .002 | | | | |
| **Symbol Digit**  **Modality Test** | | Regressions | | | Meta-analysis | |
| Studies (4) | N | β | | p | Fisher’s z (95% CI) | Weight |
| HRS | 2,722 | -.063 | | <.001 | -.063 (-.101, -.025) | 26.13 |
| ELSA | 1,009 | -.149 | | <.001 | -.150 (-.212, -.088) | 22.62 |
| Mex-Cog | 2,828 | -.036 | | .011 | -.036 (-.073, .001) | 26.22 |
| Chile-Cog | 1,846 | -.166 | | <.001 | -.168 (-.213, -.122) | 25.04 |
| Overall |  | Estimate = -.102 (-.165, -.038), p = .002 | | | | |
| Heterogeneity |  | | Q = 24.86, I^2^ (%) = 88.07, tau^2^ = .004 | | | |
| **Backward Counting or Naming** | | Regressions | | | Meta-analysis | |
| Studies (5) | N | β | | p | Fisher’s z (95% CI) | Weight |
| HRS | 2,807 | -.055 | | .001 | -.055 (-.092, -.018) | 23.90 |
| ELSA | 1,052 | -.126 | | <.001 | -.127 (-.187, -.066) | 17.13 |
| DS-HAALSI | 630 | -.065 | | .066 | -.065 (-.143, .013) | 13.13 |
| Mex-Cog | 3,069 | -.057 | | .001 | -.057 (-.092, -.022) | 24.39 |
| Chile-Cog | 1,897 | -.137 | | <.001 | -.138 (-.183, -.093) | 21.46 |
| Overall |  | Estimate = -.087 (-.124, -.050), p <.001 | | | | |
| Heterogeneity |  | Q = 12.12, I^2^ (%) = 67.07, tau^2^ = .001 | | | | |

(continues)

| **Trail Making A** | | Regressions | | | Meta-analysis |  |
| --- | --- | --- | --- | --- | --- | --- |
| Studies (2) | N | β | | p | Fisher’s z (95% CI) | Weight |
| HRS | 2765 | -.040 | | .022 | -.040 (-.077, -.003) | 67.50 |
| ELSA | 1019 | -.082 | | .008 | -.082 (-.144, -.021) | 32.50 |
| Overall |  | Estimate = -.054 (-.092, -.015), p = .006 | | | | |
| Heterogeneity |  | Q = 1.32, I^2^ (%) = 24.27, tau^2^ = .000 | | | | |
| **Trail Making B** | | Regressions | | | Meta-analysis |  |
| Studies (2) | N | β | | p | Fisher’s z (95% CI) | Weight |
| HRS | 2692 | -.085 | | <.001 | -.085 (-.123, -.047) | 74.89 |
| ELSA | 904 | -.120 | | <.001 | -.121 (-.186, -.055) | 25.11 |
| Overall |  | Estimate = -.094 (-.127, -.061), p <.001 | | | | |
| Heterogeneity |  | Q = .84, I^2^ (%) = .06, tau^2^ = .000 | | | | |
| **Constructional Praxes** | | Regressions | | | Meta-analysis |  |
| Studies (6) | N | β | | p | Fisher’s z (95% CI) | Weight |
| HRS | 2,807 | -.026 | | .141 | -.026 (-.063, .011) | 18.93 |
| ELSA | 1,048 | -.104 | | .001 | -.104 (-.165, -.044) | 14.64 |
| LASI-DAD | 3,662 | -.053 | | <.001 | -.053 (-.085 ,-.021) | 19.73 |
| DS-HAALSI | 479 | -.051 | | .177 | -.051 (-.141, .039) | 10.22 |
| Mex-Cog | 3,032 | -.042 | | .009 | -.042 (-.078, -.006) | 19.18 |
| Chile-Cog | 1,823 | -.147 | | <.001 | -.148 (-.194, -.102) | 17.30 |
| Overall |  | Estimate = -.070 (-.108, -.031), p < .001 | | | | |
| Heterogeneity |  | Q =20.55, I^2^ (%) = 77.36, tau^2^ = .002 | | | | |
| **Constructional Praxes, Delayed Recall** | | Regressions | | | Meta-analysis | |
| Studies (6) | N | β | | p | Fisher’s z (95% CI) | Weight |
| HRS | 2,806 | -.050 | | .004 | -.050 (-.087, -.013) | 20.54 |
| ELSA | 992 | -.068 | | .024 | -.068 (-.130, -.006) | 12.29 |
| LASI-DAD | 3,637 | -.059 | | <.001 | -.059 (-.092, -.027) | 22.41 |
| DS-HAALSI | 482 | -.007 | | .866 | -.007 (-.097, .083) | 7.41 |
| Mex-Cog | 2,802 | -.004 | | .786 | -.004 (-.041, .033) | 20.53 |
| Chile-Cog | 1,751 | -.094 | | <.001 | -.094 (-.141, -.047) | 16.83 |
| Overall |  | Estimate = -.049 (-.077,-.021), p <.001 | | | | |
| Heterogeneity |  | | Q =10.82, I^2^ (%) = 54.80, tau^2^ = .001 | | | |
| **Raven’s Matrixes** | | Regressions | | | Meta-analysis | |
| Studies (4) | N | β | | p | Fisher’s z (95% CI) | Weight |
| HRS | 2,793 | -.066 | | <.001 | -.066 (-.103, -.029) | 33.98 |
| ELSA | 1,053 | -.115 | | <.001 | -.116 (-.176, -.055) | 12.79 |
| LASI-DAD | 3,776 | -.085 | | <.001 | -.085 (-.117, -.053) | 45.93 |
| DS-HAALSI | 602 | -.084 | | .028 | -.084 (-.164, -.004) | 7.30 |
| Overall |  | Estimate = -.083 (.104, -.061), p <.001 | | | | |
| Heterogeneity |  | Q =1.92, I^2^ (%) = .07, tau^2^ = .000 | | | | |

(continues)

| **Jorm IQ-CODE** | | Regressions | | | Meta-analysis |  |
| --- | --- | --- | --- | --- | --- | --- |
| Studies (5) | N | β | | p | Fisher’s z (95% CI) | Weight |
| HRS | 2,149 | .103 | | <.001 | .103 (.061, .146) | 18.45 |
| ELSA | 897 | .097 | | .005 | .097 (.032, .163) | 9.41 |
| LASI-DAD | 3,957 | .089 | | <.001 | .089 (.058, .120) | 26.88 |
| CHARLS | 8,833 | .129 | | <.001 | .130 (.109, .151) | 38.38 |
| DS-HAALSI | 624 | .112 | | .007 | .112 (.034, .191) | 6.87 |
| Overall |  | Estimate = .110 (.088,.132), p <.001 | | | | |
| Heterogeneity |  | Q = 5.10, I^2^ (%) = 35.69, tau^2^ = .000 | | | | |
| **CSI-D** | | Regressions | | | Meta-analysis |  |
| Studies (7) | N | β | | p | Fisher’s z (95% CI) | Weight |
| HRS | 2,587 | .140 | | <.001 | .141 (.102, .179) | 13.33 |
| ELSA | 900 | .136 | | <.001 | .137 (.071, .202) | 5.10 |
| LASI-DAD | 3,958 | .157 | | <.001 | .158 (.127, .189) | 18.98 |
| CHARLS | 8,808 | .143 | | <.001 | .144 (.123, .165) | 33.90 |
| DS-HAALSI | 625 | .228 | | <.001 | .232 (.153, .311) | 3.59 |
| Mex-Cog | 3,172 | .149 | | <.001 | .150 (.115, .185) | 15.84 |
| Chile-Cog | 1,712 | .200 | | <.001 | .203 (.155, .250) | 9.26 |
| Overall |  | Estimate = .156 (.140, .171), p <.001 | | | | |
| Heterogeneity |  | Q = 9.56, I^2^ (%) = 14.87, tau^2^ = .000 | | | | |
| **Blessed Dementia Scale,**  **Part I** | | Regressions | | | Meta-analysis | |
| Studies (3) | N | β | | p | Fisher’s z (95% CI) | Weight |
| HRS | 2,587 | .156 | | <.001 | .157 (.119, .196) | 62.99 |
| ELSA | 899 | .137 | | <.001 | .138 (.072, .203) | 21.85 |
| DS-HAALSI | 625 | .213 | | <.001 | .216 (.138, .295) | 15.17 |
| Overall |  | Estimate = .162 (.131,.193), p <.001 | | | | |
| Heterogeneity |  | | Q = 2.41, I^2^ (%) = .01, tau^2^ = .000 | | | |
| **10/66** | | Regressions | | | Meta-analysis | |
| Studies (4) | N | β | | p | Fisher’s z (95% CI) | Weight |
| HRS | 2,587 | .159 | | <.001 | .160 (.122, .199) | 31.52 |
| ELSA | 899 | .142 | | <.001 | .143 (.077, .208) | 17.95 |
| LASI_DAD | 3,957 | .125 | | <.001 | .126 (.094, .157) | 36.61 |
| DS-HAALSI | 625 | .223 | | <.001 | .227 (.148, .305) | 13.91 |
| Overall |  | Estimate = .154 (.120, .188), p <.001 | | | | |
| Heterogeneity |  | Q = 6.24, I^2^ (%) = 49.72, tau^2^ = .000 | | | | |

*Note.* Studies are the Health and Retirement Study (HRS), the English Longitudinal Study of Ageing (ELSA), the Diagnostic Assessment of Dementia for the Longitudinal Aging Study in India (LASI-DAD), China Health and Retirement Longitudinal Study (CHARLS), Dementia Study of the Health and Aging in Africa (DS-HAALSI), the Mexican Cognitive Aging Ancillary Study (Mex-Cog) of the Mexican Health and Aging Study, and the Chile Cognitive Aging Study (Chile-Cog). N is the number of participants with data on the cognitive measures*.* βs are standardized coefficients from regressions within each sample; for informant measures, higher scores indicate cognitive decline. We further report Fisher’s z (95% Confidence Intervals) and weights for each study included in the meta-analysis. The analyses accounted for age, sex, education, marital status, and race and ethnicity (where possible).

Table S8

*Associations with five cognitive domains controlling for depressive symptoms*

| **Episodic Memory** | | Regressions | | Meta-analysis | |
| --- | --- | --- | --- | --- | --- |
| Studies (7) |  | β | p | Fisher’s z (95% CI) | Weight |
| HRS |  | -.022 | .216 | -.022 (-.059, .015) | 12.79 |
| ELSA |  | -.039 | .196 | -.039 (-.099, .021) | 4.80 |
| LASI-DAD |  | -.016 | .307 | -.016 (-.047, .015) | 18.04 |
| CHARLS |  | -.035 | .002 | -.035 (-.056, -.014) | 38.20 |
| DS-HAALSI |  | .010 | .802 | .010 (-.068, .088) | 2.84 |
| Mex-Cog |  | -.026 | .135 | -.026 (-.061, .009) | 14.43 |
| Chile-Cog |  | -.073 | .003 | -.073 (-.117, -.029) | 8.90 |
| Overall | | Estimate = -.031 (-.044, -.018), p <.001 | | | |
| Heterogeneity | | Q = 5.95, I^2^(%) = 0.02, tau^2^ = .000 | | | |
| **Speed-Attention** | | Regressions | | Meta-analysis | |
| Studies (6) |  | β | p | Fisher’s z (95% CI) | Weight |
| HRS |  | -.052 | .002 | -.052 (-.089, -.015) | 19.90 |
| ELSA |  | -.112 | <.001 | -.112 (-.173, -.052) | 11.81 |
| LASI-DAD |  | -.028 | .047 | -.028 (-.059, .003) | 22.46 |
| DS-HAALSI |  | -.084 | .033 | -.084 (-.162, -.006) | 8.16 |
| Mex-Cog |  | -.025 | .094 | -.025 (-.060, .010) | 20.85 |
| Chile-Cog |  | -.076 | .001 | -.223 (-.267, -.178) | 16.83 |
| Overall | | Estimate = -.055 (-.080, -.029), p <.001 | | | |
| Heterogeneity | | Q = 10.05, I^2^(%) = 52.00, tau^2^ = .000 | | | |
| **Visuospatial Ability** | | Regressions | | Meta-analysis | |
| Studies (6) |  | β | p | Fisher’s z (95% CI) | Weight |
| HRS |  | -.044 | .009 | -.044 (-.081, -.007) | 21.12 |
| ELSA |  | -.100 | .001 | -.100 (-.161, -.040) | 7.92 |
| LASI-DAD |  | -.057 | <.001 | -.057 (-.088, -.026) | 29.53 |
| DS-HAALSI |  | -.023 | .571 | -.023 (-.103, .057) | 4.51 |
| Mex-Cog |  | -.025 | .146 | -.025 (-.061, .011) | 22.75 |
| Chile-Cog |  | -.032 | .194 | -.032 (-.077, .013) | 14.16 |
| Overall | | Estimate = -.045 (-.062, -.028), p <.001 | | | |
| Heterogeneity | | Q = 5.62, I2(%) = 0.01, tau2 = .000 | | | |

(continues)

| **Num. Reasoning** | | Regressions | | Meta-analysis | |
| --- | --- | --- | --- | --- | --- |
| Studies (2) |  | β | p | Fisher’s z (95% CI) | Weight |
| HRS |  | -.060 | .001 | -.060 (-.100, -.020) | 71.36 |
| ELSA |  | -.072 | .023 | -.072 (-.135, -.010) | 28.64 |
| Overall | | Estimate = -.064 (-.097, -.030), p <.001 | | | |
| Heterogeneity | | Q = 0.10, I^2^(%) = .04, tau^2^ = .000 | | | |
| **Verbal Fluency** | | Regressions | | Meta-analysis | |
| Studies (7) |  | β | p | Fisher’s z (95% CI) | Weight |
| HRS |  | -.020 | .278 | -.020 (-.057, .017) | 15.30 |
| ELSA |  | -.055 | .083 | -.055 (-.115, .005) | 7.67 |
| LASI-DAD |  | .003 | .854 | .003 (-.028, .034) | 18.42 |
| CHARLS |  | .003 | .818 | .003 (-.019, .025) | 25.13 |
| DS-HAALSI |  | .034 | .461 | .034 (-.044, .112) | 4.94 |
| Mex-Cog |  | -.031 | .085 | -.031 (-.066, .004) | 16.44 |
| Chile-Cog |  | -.052 | .036 | -.052 (-.096, -.008) | 12.11 |
| Overall | | Estimate = -.016 (-.034, .003), p =.099 | | | |
| Heterogeneity | | Q = 10.50, I^2^(%) = 40.22, tau^2^ = .000 | | | |

*Note.* Studies are the Health and Retirement Study (HRS), the English Longitudinal Study of Ageing (ELSA), the Diagnostic Assessment of Dementia for the Longitudinal Aging Study in India (LASI-DAD), China Health and Retirement Longitudinal Study (CHARLS), Dementia Study of the Health and Aging in Africa (DS-HAALSI), the Mexican Cognitive Aging Ancillary Study (Mex-Cog) of the Mexican Health and Aging Study, and the Chile Cognitive Aging Study (Chile-Cog). βs are standardized coefficients from regressions within each sample. We further report Fisher’s z (95% Confidence Intervals) and weights for each study included in the meta-analysis. Results controlling for age, sex, education, marital status, and race and ethnicity (where possible) are reported in Table 2; the analysis reported here further accounted for depressive symptoms.

Table S9

*Associations with five cognitive domains controlling for living arrangements*

| **Episodic Memory** | | Regressions | | Meta-analysis | |
| --- | --- | --- | --- | --- | --- |
| Studies (7) |  | β | p | Fisher’s z (95% CI) | Weight |
| HRS |  | -.042 | .011 | -.042 (-.079, -.005) | 17.98 |
| ELSA |  | -.054 | .058 | -.054 (-.114, .006) | 10.69 |
| LASI-DAD |  | -.070 | <.001 | -.070 (-.101, -.039) | 20.40 |
| CHARLS |  | -.097 | <.001 | -.097 (-.119, -.076) | 24.69 |
| DS-HAALSI |  | -.047 | .200 | -.047 (-.125, .031) | 7.39 |
| Mex-Cog |  | -.032 | .040 | -.032 (-.067, .003) | 18.85 |
| Chile-Cog |  | -- |  | -- | -- |
| Overall | | Estimate = -.061 (-.086, -.037), p <.001 | | | |
| Heterogeneity | | Q = 13.70, I^2^(%) = 60.30, tau^2^ = .001 | | | |
| **Speed-Attention** | | Regressions | | Meta-analysis | |
| Studies (6) |  | β | p | Fisher’s z (95% CI) | Weight |
| HRS |  | -.080 | <.001 | -.080 (-.117, -.043) | 24.54 |
| ELSA |  | -.144 | <.001 | -.145 (-.205, -.085) | 10.70 |
| LASI-DAD |  | -.068 | <.001 | -.068 (-.099, -.037) | 31.29 |
| DS-HAALSI |  | -.106 | .002 | -.106 (-.185, -.028) | 6.61 |
| Mex-Cog |  | -.065 | <.001 | -.065 (-.100, -.030) | 26.86 |
| Chile-Cog |  | -- |  | -- | -- |
| Overall | | Estimate = -.081 (-.102, -.060), p < .001 | | | |
| Heterogeneity | | Q = 6.16, I^2^(%) = 17.95, tau^2^ = .000 | | | |
| **Visuospatial Ability** | | Regressions | | Meta-analysis | |
| Studies (6) |  | β | p | Fisher’s z (95% CI) | Weight |
| HRS |  | -.063 | <.001 | -.063 (-.100, -.026) | 23.90 |
| ELSA |  | -.114 | <.001 | -.114 (-.175, -.054) | 14.74 |
| LASI-DAD |  | -.093 | <.001 | -.093 (-.125, -.062) | 26.74 |
| DS-HAALSI |  | -.074 | .037 | -.074 (-.154, .006) | 10.06 |
| Mex-Cog |  | -.031 | .044 | -.031 (-.067, .005) | 24.56 |
| Chile-Cog |  | -- |  | -- | -- |
| Overall | | Estimate = -.072 (-.102, -.042), p <.001 | | | |
| Heterogeneity | | Q = 8.97, I2(%) = 55.80, tau2 = .001 | | | |

(continues)

| **Num. Reasoning** | | Regressions | | Meta-analysis | |
| --- | --- | --- | --- | --- | --- |
| Studies (2) |  | β | p | Fisher’s z (95% CI) | Weight |
| HRS |  | -.088 | <.001 | -.088 (-.128, -.049) | 71.36 |
| ELSA |  | -.116 | <.001 | -.117 (-.179, -.054) | 28.64 |
| Overall | | Estimate = -.096 (-.130, -.063), p <.001 | | | |
| Heterogeneity | | Q = 0.56, I^2^(%) = .07, tau^2^ = .000 | | | |
| **Verbal Fluency** | | Regressions | | Meta-analysis | |
| Studies (7) |  | β | p | Fisher’s z (95% CI) | Weight |
| HRS |  | -.040 | .022 | -.040 (-.077, -.003) | 14.25 |
| ELSA |  | -.075 | .011 | -.075 (-.135, -.015) | 5.34 |
| LASI-DAD |  | -.043 | .005 | -.043 (-.074, -.012) | 19.89 |
| CHARLS |  | -.064 | <.001 | -.064 (-.086, -.042 | 41.25 |
| DS-HAALSI |  | -.036 | .379 | -.036 (-.114, .042) | 3.16 |
| Mex-Cog |  | -.049 | .003 | -.049 (-.084, -.014) | 16.12 |
| Chile-Cog |  | -- |  | -- | -- |
| Overall | | Estimate = -.054 (-.068, -.040), p <.001 | | | |
| Heterogeneity | | Q = 2.61, I^2^(%) = 0.01, tau^2^ = .000 | | | |

*Note.* Studies are the Health and Retirement Study (HRS), the English Longitudinal Study of Ageing (ELSA), the Diagnostic Assessment of Dementia for the Longitudinal Aging Study in India (LASI-DAD), China Health and Retirement Longitudinal Study (CHARLS), Dementia Study of the Health and Aging in Africa (DS-HAALSI), the Mexican Cognitive Aging Ancillary Study (Mex-Cog) of the Mexican Health and Aging Study, and the Chile Cognitive Aging Study (Chile-Cog). βs are standardized coefficients from regressions within each sample. We further report Fisher’s z (95% Confidence Intervals) and weights for each study included in the meta-analysis. Results controlling for age, sex, education, marital status, and race and ethnicity (where possible) are reported in Table 2; the analysis reported here further accounted for living arrangements (i.e., living alone). Information on living arrangements was not retrieved for the Chile Cognitive Aging Study (Chile-Cog) and thus the study was not included in this follow-up analysis.

Table S10

*Meta-analysis of interactions*

|  | Interaction with… | | | | | | | | | | | | | | | |
| --- | --- | --- | --- | --- | --- | --- | --- | --- | --- | --- | --- | --- | --- | --- | --- | --- |
| **Overall** | Depression | | Cog. Imp. | | Age | | Sex | | Education | | Mar. Status | | Liv. Alone | | Race | |
| **Cognition** | β | p | β | p | β | p | β | p | β | p | β | p | β | p | β | p |
| HRS | .039 | .048 | -.008 | .471 | -.026 | .119 | .010 | .722 | -.006 | .707 | .013 | .547 | -.010 | .615 | -.007 | .722 |
| ELSA | .028 | .381 | .025 | .269 | .006 | .824 | -.011 | .799 | .032 | .261 | -.031 | .456 | .038 | .330 | -.034 | .230 |
| LASI-DAD | .007 | .645 | -.007 | .534 | -.022 | .094 | -.006 | .773 | .065 | <.001 | .032 | .098 | .012 | .437 | -- |  |
| CHARLS | .009 | .352 | .038 | <.001 | -.012 | .153 | -.041 | .001 | .035 | <.001 | -.046 | .001 | .035 | <.001 | -- |  |
| DS-HAALSI | -.092 | .006 | .015 | .619 | .015 | .631 | -.032 | .511 | .007 | .824 | -.068 | .070 | .030 | .413 | -- |  |
| Mex-Cog | .017 | .319 | .002 | .883 | -.033 | .023 | -.034 | .146 | .039 | .008 | .048 | .011 | -.008 | .640 | -- |  |
| Chile-Cog | .022 | .356 | .022 | .202 | .009 | .666 | .082 | .016 | -- |  | -- |  | -- |  | -- |  |
| Meta-analysis | Est.= .013 (95% CI = -.000,.026), p = .054 | | Est.= .013 (95% CI = -.005,.031), p = .162 | | Est.=-.015 (95% CI =-.028,-.002), p = .022 | | Est.=-.005(95% CI =-.037,.027), p = .773 | | Est.= .036 (95% CI =.018,.053),  p < .001 | | Est.=-.004(95% CI =-.040,.032),  p = .832 | | Est.=.015 (95% CI =-.005,.034),  p = .137 | | Est.= -.014 (95% CI = -.046,.017), p = .370 | |
| **Informant** | Depression | | Cog. Imp. | | Age | | Sex | | Education | | Mar. Status | | Liv. Alone | | Race | |
| **ratings** | β | p | β | p | β | p | β | p | β | p | β | p | β | p | β | p |
| HRS | -.006 | .774 | -.003 | .884 | .018 | .337 | .039 | .201 | -.011 | .554 | -.030 | .299 | -.018 | .412 | .031 | .133 |
| ELSA | -.058 | .120 | .006 | .854 | -.015 | .640 | .024 | .640 | -.002 | .941 | .040 | .408 | -.062 | .152 | .021 | .516 |
| LASI-DAD | -.015 | .329 | .002 | .924 | .010 | .524 | -.006 | .793 | -.026 | .090 | -.002 | .928 | -.009 | .611 | -- |  |
| CHARLS | -.029 | .017 | -.027 | .019 | -.004 | .676 | .034 | .031 | .016 | .135 | .031 | .085 | -.020 | .104 | -- |  |
| DS-HAALSI | .159 | <.001 | -.002 | .961 | -.027 | .498 | .180 | .006 | -.012 | .770 | .022 | .630 | -.145 | .002 | -- |  |
| Mex-Cog | -.011 | .583 | .007 | .701 | -.023 | .183 | .069 | .014 | -.035 | .046 | .001 | .954 | .007 | .703 | -- |  |
| Chile-Cog | .032 | .236 | -.032 | .219 | -.047 | .045 | .042 | .287 | -- |  | -- |  | -- |  | -- |  |
| Meta-analysis | Est = .005 (95% CI = -.038,.048),  p = .814 | | Est =-.011 (95% CI =-.026,.005),  p = .175 | | Est =-.006 (95% CI =-.019,.007),  p = .368 | | Est =.048(95% CI = .013,.083), p = .007 | | Est =-.010 (95% CI =-.031,.011), p = .333 | | Est = .008(95% CI =-.014,.030), p = .467 | | Est = -.030(95% CI =-.062,.002), p = .067 | | Est = .028 (95% CI = -.005,.062), p = .093 | |

(continues)

|  | Interaction with… | | | | | | | | | | | | | | | |
| --- | --- | --- | --- | --- | --- | --- | --- | --- | --- | --- | --- | --- | --- | --- | --- | --- |
| **Episodic** | Depression | | Cog. Imp. | | Age | | Sex | | Education | | Mar. Status | | Liv. Alone | | Race | |
| **Memory** | β | p | β | p | β | p | β | p | β | p | β | p | β | p | β | p |
| HRS | .027 | .148 | .006 | .724 | -.001 | .961 | .009 | .731 | .006 | .710 | .012 | .622 | .014 | .474 | .008 | .662 |
| ELSA | -.005 | .862 | .023 | .378 | .004 | .874 | -.028 | .508 | .000 | .986 | -.001 | .973 | .002 | .966 | .015 | .587 |
| LASI-DAD | .004 | .792 | .018 | .203 | -.015 | .257 | .001 | .947 | .016 | .240 | -.018 | .392 | .034 | .036 | -- |  |
| CHARLS | -.006 | .607 | .032 | .001 | -.012 | .193 | -.028 | .049 | .030 | .002 | -.066 | <.001 | .046 | <.001 | -- |  |
| DS-HAALSI | -.069 | .079 | -.013 | .735 | .012 | .749 | -.039 | .488 | -.038 | .305 | -.045 | .304 | .017 | .678 | -- |  |
| Mex-Cog | .028 | .122 | .028 | .084 | .004 | .795 | .005 | .835 | .004 | .794 | .008 | .690 | -.035 | .044 | -- |  |
| Chile-Cog | .049 | .039 | .014 | .519 | .003 | .878 | .015 | .648 | -- |  | -- |  |  |  | -- |  |
| Meta-analysis | Est.= .010 (95% CI =-.008,.028),  p = .278 | | Est.= .022(95% CI =.009,.035), p < .001 | | Est.= -.006(95% CI =-.019,.007), p = .369 | | Est.= -.008(95% CI =-.024,.009), p = .367 | | Est.= .015(95% CI =.000,.030), p = .045 | | Est.= -.019(95% CI =-.048,.010), p = .201 | | Est.= .015(95% CI =-.012,.042), p = .276 | | Est.= .010(95% CI =-.022,.041), p = .537 | |
| **Speed** | Depression | | Cog. Imp. | | Age | | Sex | | Education | | Mar. Status | | Liv. Alone | | Race | |
| **Attention** | β | p | β | p | β | p | β | p | β | p | β | p | β | p | β | p |
| HRS | .027 | .127 | .002 | .909 | -.008 | .596 | -.009 | .704 | -.011 | .460 | -.004 | .865 | -.009 | .634 | -.005 | .751 |
| ELSA | .019 | .512 | .062 | .012 | .013 | .617 | .000 | .995 | .000 | .988 | .002 | .953 | .004 | .916 | -.015 | .570 |
| LASI-DAD | .060 | <.001 | .027 | .050 | .032 | .013 | .026 | .191 | -.015 | .237 | -.047 | .015 | .012 | .406 | -- |  |
| DS-HAALSI | .019 | .608 | -.003 | .934 | .006 | .863 | -.059 | .268 | -.021 | .558 | .000 | .996 | -.007 | .852 | -- |  |
| Mex-Cog | .034 | .028 | -.021 | .119 | -.014 | .278 | -.030 | .154 | .004 | .758 | .026 | .131 | -.016 | .272 | -- |  |
| Chile-Cog | .085 | <.001 | -.009 | .665 | .052 | .009 | .038 | .225 | -- |  | -- |  | -- |  | -- |  |
| Meta-analysis | Est.= .045 (95% CI =.025,.065), p <.001 | | Est.=.007 (95% CI =-.015,.029), p = .522 | | Est.= .013 (95% CI =-.010,.037), p = .258 | | Est.=-.001 (95% CI =-.026,.025), p = .961 | | Est.=-.008(95% CI=-.026,.010),  p = .403 | | Est.=-.007 (95% CI =-.036,.023), p = .663 | | Est.=-.003(95% CI=-.021,.016), p = .784 | | Est.=-.008(95% CI =-.039,.024), p = .630 | |

(continues)

|  | Interaction with… | | | | | | | | | | | | | | | |
| --- | --- | --- | --- | --- | --- | --- | --- | --- | --- | --- | --- | --- | --- | --- | --- | --- |
| **Visuo-spatial** | Depression | | Cog. Imp. | | Age | | Sex | | Education | | Mar. Status | | Liv. Alone | | Race |  |
| **Ability** | β | p | β | p | β | p | β | p | β | p | β | p | β | p | β | p |
| HRS | .029 | .113 | -.017 | .250 | -.015 | .312 | .034 | .174 | -.014 | .372 | -.016 | .467 | .010 | .581 | -.024 | .163 |
| ELSA | .039 | .210 | .024 | .364 | .000 | .997 | .027 | .513 | -.014 | .596 | -.043 | .266 | .050 | .183 | -.032 | .240 |
| LASI-DAD | -.020 | .151 | -.016 | .235 | .004 | .777 | -.007 | .737 | .035 | .008 | -.001 | .942 | .014 | .343 | -- |  |
| DS-HAALSI | -.028 | .463 | -.043 | .254 | -.017 | .622 | .027 | .618 | .031 | .403 | -.013 | .769 | .016 | .687 | -- |  |
| Mex-Cog | .022 | .226 | -.002 | .885 | -.023 | .120 | -.010 | .675 | .024 | .112 | .029 | .147 | -.014 | .424 | -- |  |
| Chile-Cog | .051 | .032 | -.016 | .488 | .008 | .714 | .031 | .364 | -- |  | -- |  |  |  | -- |  |
| Meta-analysis | Est.=.017 (95% CI =-.008,.041), p = .188 | | Est.=-.011(95% CI =-.028,.006), p = .201 | | Est.=-.007(95% CI =-.024,.010), p = .429 | | Est.=.012(95% CI =-008,.032),  p = .237 | | Est.=.014(95% CI =-.009,.037), p = .242 | | Est.=-.003(95% CI =-.025,.020), p = .812 | | Est.=.009(95% CI =-.009,.027), p = .335 | | Est.=-.026(95% CI =-.058,.005), p = .103 | |
| **Numeric** | Depression | | Cog. Imp. | | Age | | Sex | | Education | | Mar. Status | | Liv. Alone | | Race |  |
| **Reasoning** | β | p | β | p | β | p | β | p | β | p | β | p | β | p | β | p |
| HRS | .009 | .649 | -.011 | .517 | -.001 | .937 | -.006 | .836 | .002 | .927 | .022 | .382 | .002 | .929 | -.025 | .170 |
| ELSA | -.006 | .848 | .036 | .215 | .016 | .561 | -.022 | .621 | .014 | .624 | -.026 | .529 | .019 | .631 | -.032 | .269 |
| Meta-analysis | Est.=.005 (95% CI =-.029,.038), p = .783 | | Est.=.006 (95% CI =-.038,.050), p = .789 | | Est.=.004 (95% CI =-.030,.037), p = .821 | | Est.=-.011(95% CI =-.044,.023), p = .535 | | Est.=.005 (95% CI =-.028,.039), p = .750 | | Est.=.004(95% CI =-.041,.050), p = .851 | | Est.=.007(95% CI =-.027,.040), p = .687 | | Est.=-.027 (95% CI =-.060,.006),  p = .113 | |
| **Verbal** | Depression | | Cog. Imp. | | Age | | Sex | | Education | | Mar. Status | | Liv. Alone | | Race |  |
| **Fluency** | β | p | β | p | β | p | β | p | β | p | β | p | β | p | β | p |
| HRS | .015 | .462 | -.005 | .782 | .004 | .813 | -.024 | .388 | .010 | .573 | .012 | .634 | .012 | .568 | .010 | .573 |
| ELSA | .000 | .991 | .023 | .419 | .001 | .970 | -.032 | .476 | -.050 | .229 | .020 | .489 | .057 | .149 | .020 | .489 |
| LASI-DAD | .032 | .054 | -.008 | .616 | -.035 | .019 | .047 | .045 | .016 | .300 | .002 | .920 | .020 | .268 | -- |  |
| CHARLS | .036 | .003 | .008 | .461 | -.012 | .260 | -.027 | .087 | -.005 | .630 | -.049 | .006 | .039 | .002 | -- |  |
| DS-HAALSI | .039 | .382 | .000 | .992 | .063 | .122 | .058 | .356 | -.120 | .004 | -.059 | .228 | .023 | .631 | -- |  |
| Mex-Cog | .035 | .066 | .032 | .059 | .001 | .934 | .004 | .872 | -.012 | .454 | -.001 | .950 | -.006 | .720 | -- |  |
| Chile-Cog | .070 | .005 | -.008 | .742 | .013 | .542 | .017 | .616 | -- |  | -- |  | -- |  | -- |  |
| Meta-analysis | Est.=.034(95% CI =.021,.047), p <.001 | | Est.=.006(95% CI =-.007,.019), p = .376 | | Est.=-.006(95% CI=-.021,.009), p = .409 | | Est.=.003 (95% CI =-.023,.028), p = .821 | | Est.=-.014(95% CI =-.041,.013), p = .303 | | Est.=-.012(95% CI =-.037,.014), p = .363 | | Est.=.023(95% CI =-.005,.041), p = .011 | | Est.=.013 (95% CI =-.019,.044), p = .428 | |

*Note.* Studies are the Health and Retirement Study (HRS), the English Longitudinal Study of Ageing (ELSA), the Diagnostic Assessment of Dementia for the Longitudinal Aging Study in India (LASI-DAD), China Health and Retirement Longitudinal Study (CHARLS), Dementia Study of the Health and Aging in Africa (DS-HAALSI), the Mexican Cognitive Aging Ancillary Study (Mex-Cog) of the Mexican Health and Aging Study, and the Chile Cognitive Aging Study (Chile-Cog). Ethnicity was tested as a moderator in HRS, but none of the interactions between loneliness and this variable was found significant: Loneliness × Ethnicity, β = .01 for overall cognition and visuospatial ability, β = .00 for episodic memory, speed attention, and numeric reasoning, and β = -.02 for informant-rated decline and verbal fluency (all ps>.01).

Figure S1

*Sub-group analysis: Loneliness association with overall cognition*

*
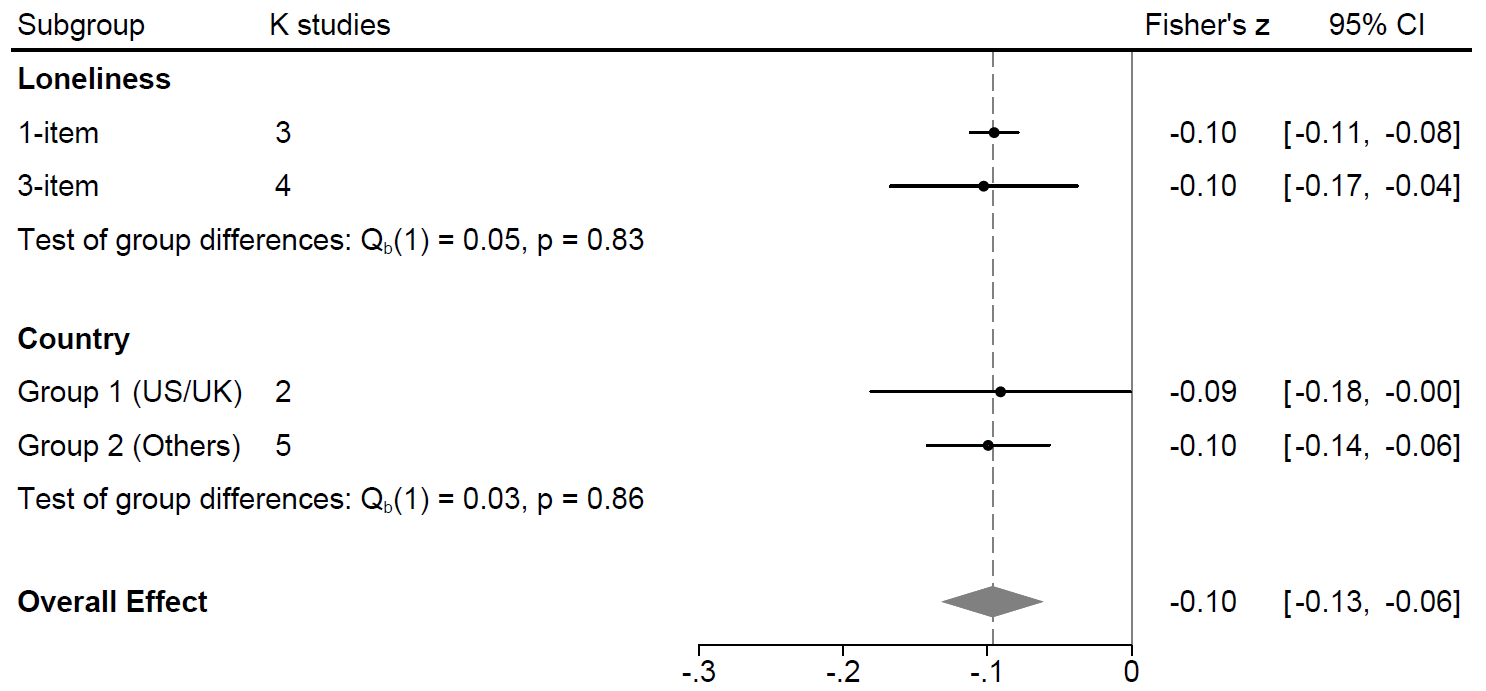
*

Figure S2

*Sub-group analysis: Loneliness association with informant-rated cognitive decline*

*
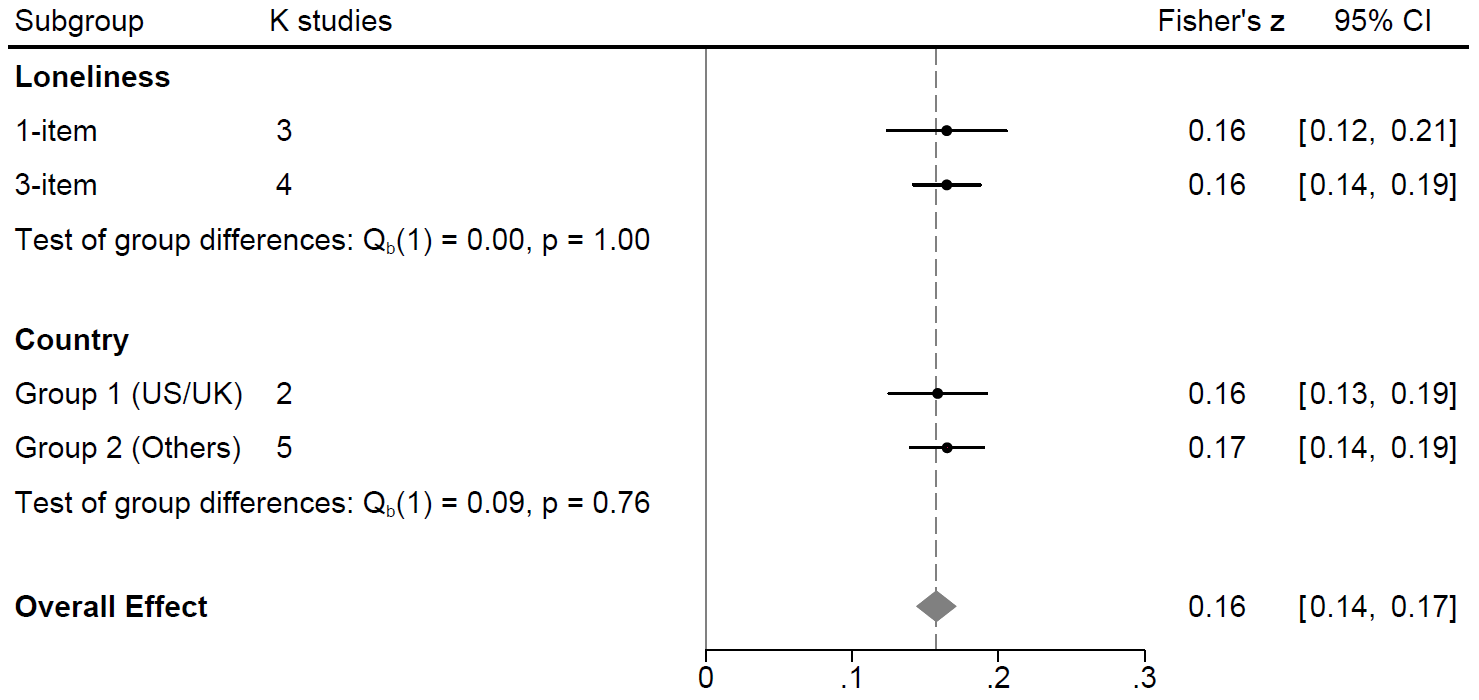
*

Figure S3

*Sub-group analysis: Loneliness association with episodic memory*


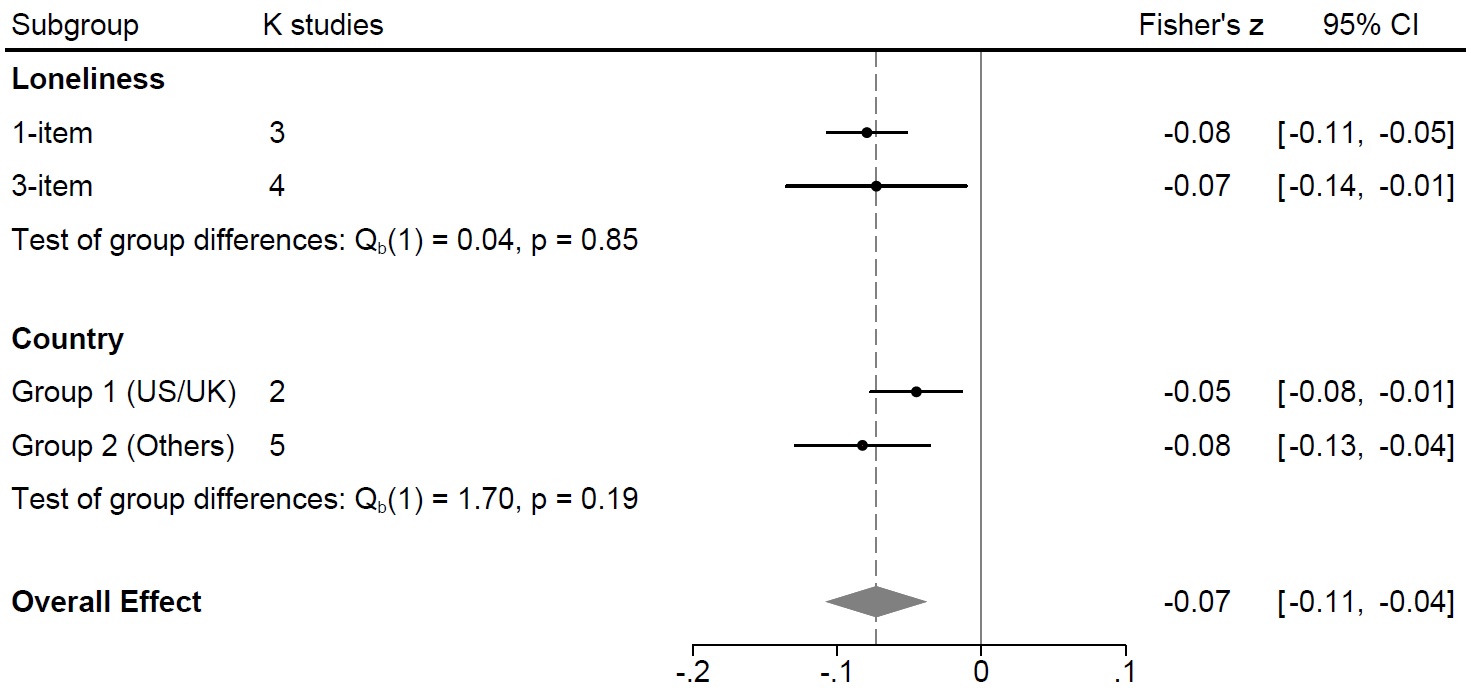


Figure S4

*Sub-group analysis: Loneliness association with speed-attention*


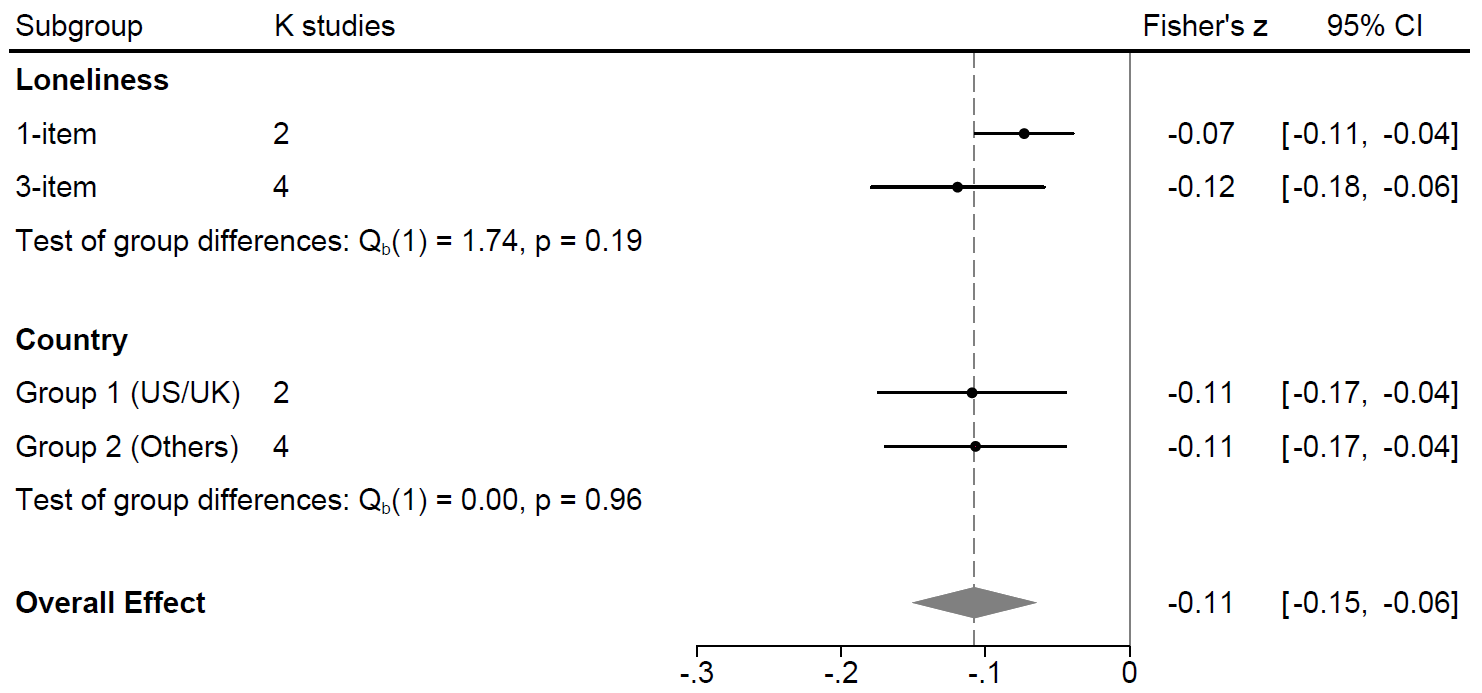


Figure S5

*Sub-group analysis: Loneliness association with visuospatial abilities*


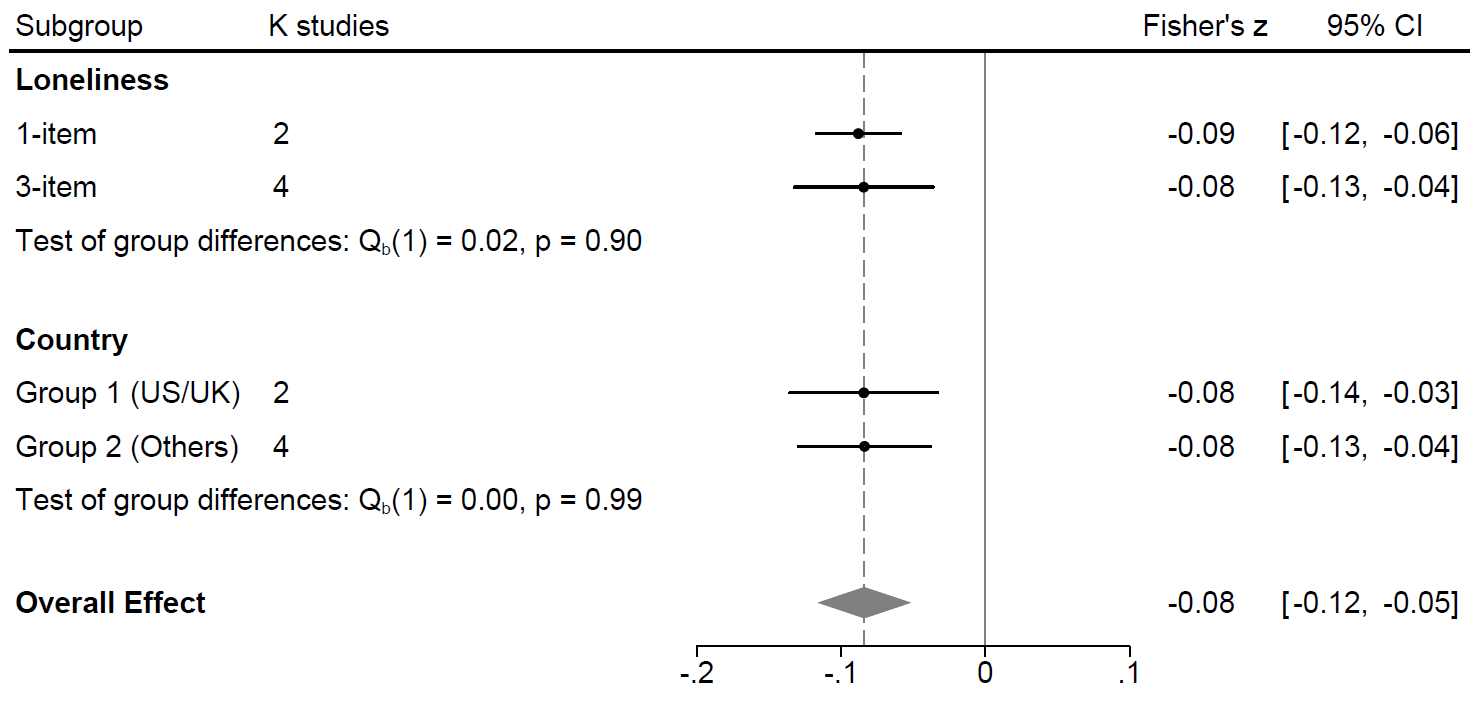


Figure S6

*Sub-group analysis: Loneliness association with verbal fluency*


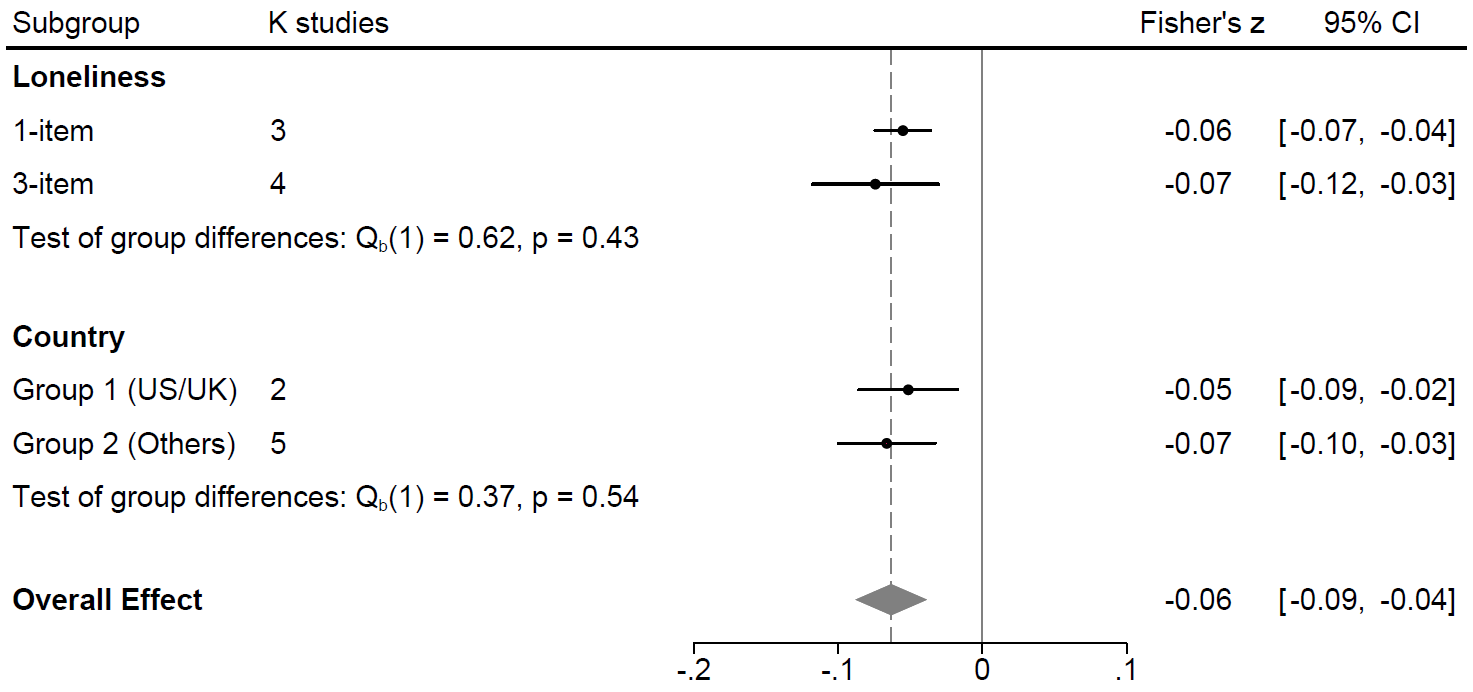


Figure S7

*Graphical representation of interactions*

**Panel (a)**

Loneliness x Depression

DV = Speed-attention


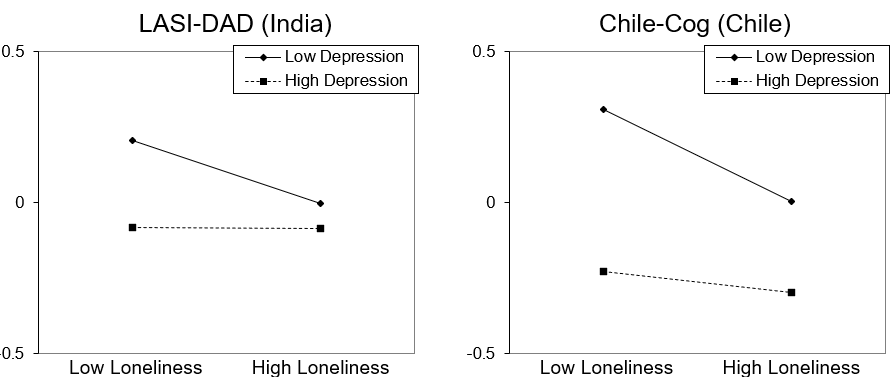


**Panel (b)**

Loneliness x Depression

DV = Verbal Fluency

**
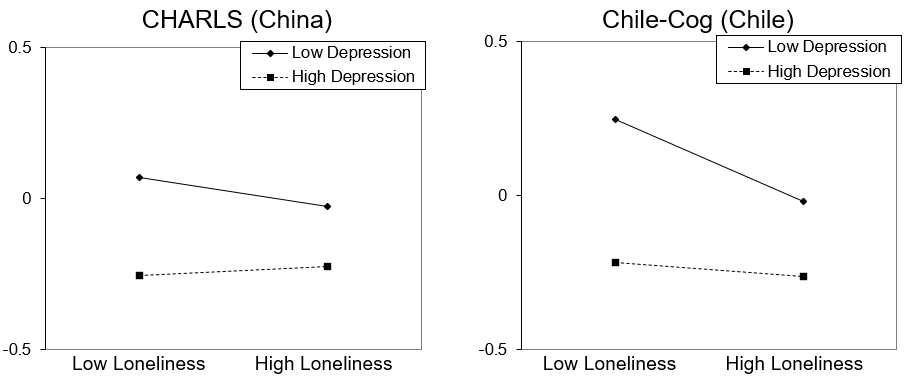
**

**Panel (c)**

Loneliness x Cognitive Impairment (Yes/No)

DV = Episodic Memory


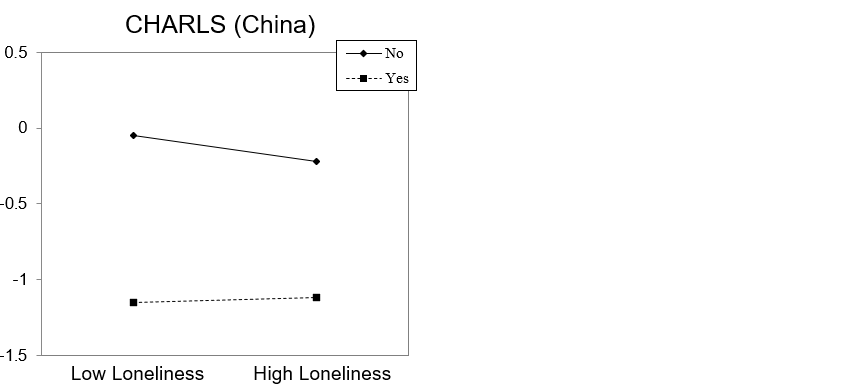


**Panel (d)**

Loneliness x Education

DV = Overall Cognition


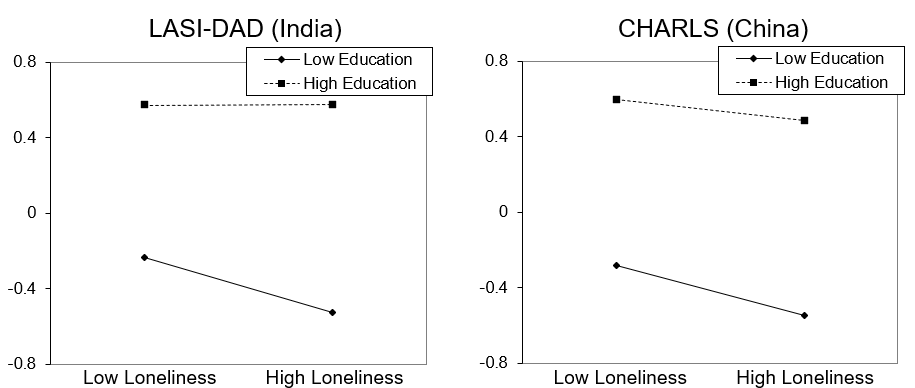


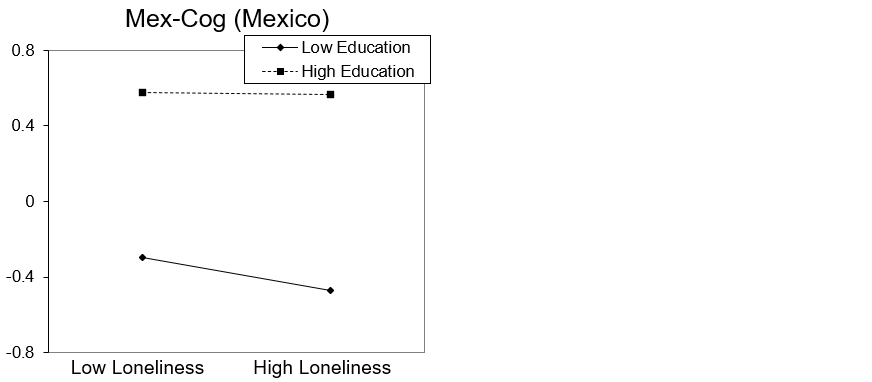


**Panel (e)**

Loneliness x Sex

DV = Informant Ratings


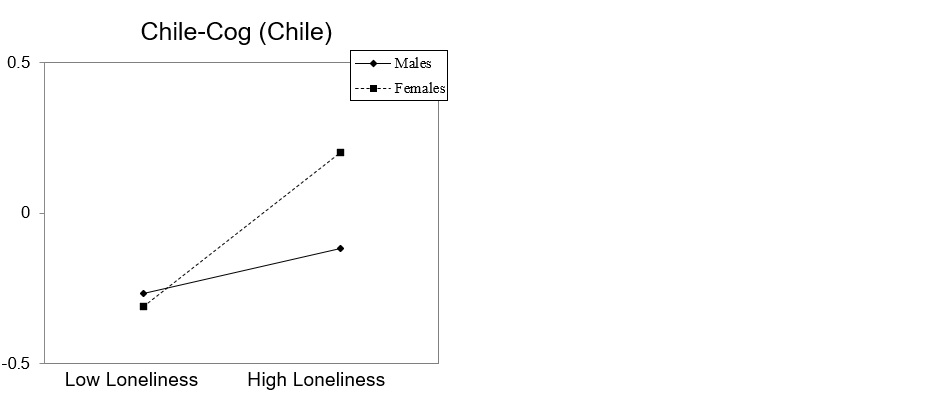


*Note.* For the meta-analysis of interactions that indicated a significant moderation, we plotted the effect for those samples for which the interaction term was significant. The graphs were generated using Excel templates from <http://www.jeremydawson.co.uk/slopes.htm>. Dawson, J. F. (2014). Moderation in management research: What, why, when and how. *Journal of Business and Psychology, 29, 1-19.*
